# Exploring the Mechanism of *Astragalus propinquus* Schischkin and *Panax Notoginseng* (A&P) Compounds in the Treatment of Renal Fibrosis and Chronic Kidney Disease Based on Integrated Network Analysis

**DOI:** 10.1155/2022/2646022

**Published:** 2022-02-27

**Authors:** Wang Xiaojia, Li Jianchun, Zhu Bingwen, He Xingcan, Deng Yanmei, Wang Li

**Affiliations:** Research Center of Integrated Traditional Chinese and Western Medicine, Affiliated Traditional Chinese Medicine Hospital of Southwest Medical University, Luzhou 646000, Sichuan, China

## Abstract

*Astragalus propinquus* Schischkin and *Panax notoginseng* (A&P) has been widely used in clinical practice to treat chronic kidney disease (CKD) for many years and achieved a remarkable improvement of these outcomes. However, its mechanisms for ameliorating CKD are still poorly obscure. In the current study, integrated network analysis was carried out to analyze the potential active ingredients and molecular mechanism of A&P on CKD, and 39 active ingredients and a total of 570 targets were obtained. Furthermore, the potential disease-related genes were obtained from the NCBI GEO database by integrating 2 microarray datasets, and 24 significant genes were utilized for subsequent analysis. Gene Ontology (GO) and Kyoto Encyclopedia of Genes and Genomes (KEGG) enrichment analysis displayed that pathways including cell oxidative stress and Akt signaling pathway are medicated by A&P. Of note, Heat Shock Transcription Factor 1 (HSF1) and RELA Proto-Oncogene (RELA) were regarded as hub genes considering their central roles in the gene regulatory network. What's more, the effect of A&P and potential genes was furthermore verified by using unilateral ureteral ligation (UUO) in rodent model. The results showed that the expression of HSF1 and RELA both at transcript and protein level was significantly upregulated in UUO model, but the expression was markedly reversed after A&P intervention. To further guide the interpretation of active ingredients from A&P on the effect of HSF1 and RELA, we performed a molecular docking assay and the results showed that active ingredients such as coptisine docked well into HSF1 and RELA. In total, these results suggest that A&P may improve RF in CKD by regulating HSF1 and RELA, which provides a basis for further understanding the mechanism of A&P in the treatment of RF and CKD.

## 1. Introduction

With the increasing incidence, morbidity and mortality of chronic kidney disease, CKD, have been recognized by the international medical community as global public health and social problem [1]. The total prevalence of CKD in the general population worldwide has reached 14.3% [2], and the number of patients with CKD worldwide has reached 697.5 million. Of note, the number of RIF patients has also shown an upward trend. It is conceivable that the figures in less-developed regions are even more alarming. In clinical practice, the main strategies to delay the progress of CKD include symptomatic treatment such as control of urinary protein, blood pressure, blood glucose, and blood lipid, correction of complications, dialysis (hemodialysis and peritoneal dialysis) [3], and end-stage renal replacement therapy of renal transplantation. However, these strategies have also shown limited success.

It is widely accepted that renal fibrosis (RF) is a final common pathological pathway of chronic kidney disease (CKD) and its progression significantly promotes renal failure. RF is characterized by the decreases in renal function, the accumulation of fibroblasts, apoptosis of renal tubular epithelial cells, and infiltration of immune cells including T cells and macrophages. Unresolved inflammation after a chronic injury caused by CKD is a continuous driving factor for renal fibrosis. The vicious circle of inflammation, tissue damage, and fibrosis increases the pressure of profibrotic cytokines, which inevitably leads to the activation of stromal-producing cells, indicating that renal fibrosis plays a vital role in the progression of CKD. It is now acknowledged that the degree of renal fibrosis is closely related to renal function and CKD [4]. Related research has demonstrated that renal fibrosis could be reversible [5]. Consequently, effective inhibition of renal fibrosis may be a pivotal strategy to prevent the progression of CKD. Currently, there is no specific treatment for CKD.

Traditional Chinese medicine (TCM) compounds, rather than a single herb, have been widely used for thousands of years for the prevention and treatment of kidney diseases. For the past few years, TCM has been an increasingly important strategy for the treatment of CKD in China due to its good therapeutic effect and low toxic side effects. In recent years, a tremendous amount of research has shown that the natural compound possesses obvious advantages, especially in the treatment of fibrosis [6]. For example, protocatechuic aldehyde (PCA), a natural phenolic acid isolated from *Salvia miltiorrhiza*, can preserve the moderate to severe deterioration of renal function [7]. In addition, isoliquiritigenin (ISL) extracted from *Glycyrrhiza uralensis* can inhibit renal fibrosis, which is also protected from kidney damage in a rodent model [8]. A broad variety of active compounds from natural sources enrich the understanding of TCM. However, TCM herb compound formula, which is often composed of different compounds, makes it difficult to ascertain its efficacy and mechanism. Network pharmacology, a recently developed and powerful tool, which integrates systems biology and polypharmacology, molecular network data, bioinformatics, and computer simulation, is well suited for TCM compound and extensively used by relevant researchers [9]. In brief, network pharmacology focuses on its active ingredients and targets in the interactome in a holistic manner to elaborate the pharmacological mechanism of drug formulas which is consistent to a certain degree with a holistic view of TCM [10].

**Figure 1:**
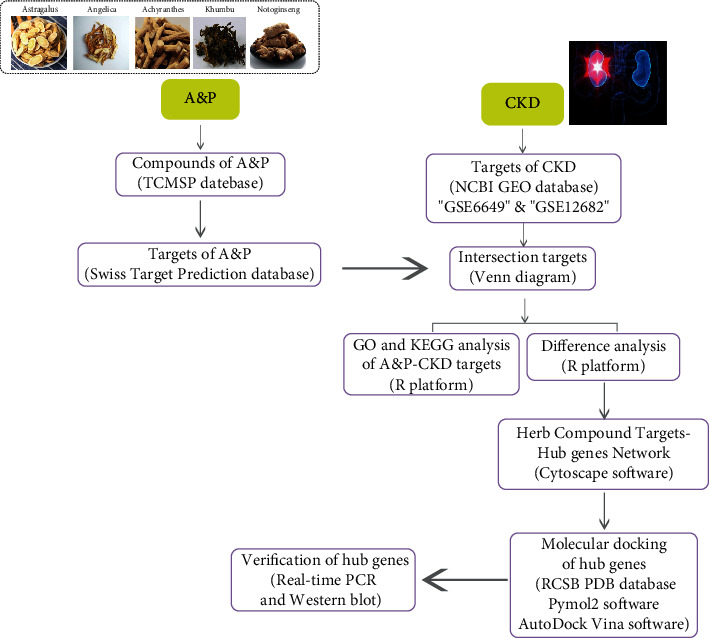
Flowchart of investigation on the mechanism of A&P in the treatment of renal fibrosis in chronic kidney disease. First, the potential active components and targets were obtained from TCMSP and Target Prediction database. Second, CKD-related microarrays on NCBI were used to obtain disease-related targets. The intersection of active ingredient targets and disease targets is regarded as a common target. Thirdly, Go and KEGG enrichment analyses were performed for common targets. The differences of common targets were analyzed and visualized; heatmap was made. Cytoscape is used to construct the regulatory network of TCM. Then, UUO model was constructed to verify the hub gene and determine the effects of A&P. PyMOL is used for molecular docking of potentially active components and hub genes.

In this article, *Astragalus propinquus* Schischkin and *Panax notoginseng* (A&P), a TCM compound preparation under the guidance of the theory of “kidney flaccidity” in traditional Chinese medicine, is composed of *Astragalus propinquus* Schischkin, *Ecklonia kurome*, *Angelica sinensis*, *Achyranthes bidentata*, *Panax notoginseng* (the formula is listed in Table 1) [11]. *Astragalus propinquus* Schischkin and *Panax notoginseng* play a major role in compound prescriptions as monarch drugs [12]. These two Chinese herbal medicines have been extensively studied for their powerful pharmacological effects and pharmaceutical value. *Astragalus* is widely grown in Northeast China, North China, and Northwest China, as well as Mongolia and Korea [13]. It has good immunomodulatory activity, anti-inflammatory activity, antioxidant activity, and antiviral activity [14]. *Panax notoginseng* has been used for four centuries. It is a precious traditional Chinese medicine with a history of more than 400 years for medical purposes. It has been extensively studied for its antitumor [15], antioxidant, antiphotoaging, anti-inflammatory, antidiabetic, and neuroprotective activities [16]. Previous studies including clinical studies and preclinically experimental investigations on A&P revealed that A&P could significantly improve the total effective rate and quality of life of patients with stage III chronic kidney disease [17]. However, considering the sophisticated action characteristics of TCM, it has brought many impediments to scientific interpretation of the efficacy characteristics of A&P and the process of disease prevention and remedy. In the present study, we primarily elaborated the potential mechanism of A&P by identifying its active ingredients and its CKD-related targets by network pharmacology. Furthermore, these results were validated by experimental studies, where we determined the protective effect of A&P in UUO in a rodent model. What's more, a molecular docking assay was performed to evaluate the hub genes and its targets interaction (the detailed flowchart is listed in Figure 1).

## 2. Materials and Methods

### 2.1. Identification of Active Ingredients of A&P and Their Potential Targets

In clinical practice, oral administration is the main route in the of administration for TCM. Oral bioavailability (OB) and drug likeness (DL) are important pharmacokinetic parameters in the drug's ADME related to absorption, distribution, and metabolism [18]. Hence, we manually retrieved the active ingredients of A&P on the platform, the Traditional Chinese Medicine Systems Pharmacology (TCMSP, https://www.tcmspw.com/tcmsp.php) by using the keywords “huangqi”, “sanqi” “danggui”, “niuxi”, “kunbu” [19]. Furthermore, the active ingredients were filtered with the parameters of *OB* ≥ 30% and DL ≥ 0.18. Immediately thereafter, the targets of potential active ingredients were performed on the Swiss Target Prediction database (The inclusion criteria are as follows: the species is “*Homo sapiens*” and the probability ≥0.6) [20] and TCMSP. Finally, the targets both in the Swiss Target Prediction database and TCMSP were regarded as the real targets of potential active ingredients.

### 2.2. Acquisition of CKD-Related Targets

The microarray dataset was retrieved with the keyword “CKD” and the number of samples per group ≥20 on the NCBI GEO database (https://www.ncbi.nlm.nih.gov/geo/). A total of 2 sets of microarrays was obtained; The R package “limma” is applied for background correction and normalization when processing with the original data. We also integrated the female tubulointerstitial and male tubulointerstitial group as the model group and sham is used as the control group. The differentially expressed genes between the model group and the control group were considered as CKD-related targets.

### 2.3. Construction of A&P-Active Ingredients-Targets Intergated Network

On the above basis, we further aim to construct a drug-target-disease molecular network. In order to obtain more accurate targets for A&P intervention in CKD, the cut-off criteria were set as follows: |logFC|> 1and *p*-vaule <0.1. Then, the differential genes were obtained by intersecting the targets of active ingredients in A&P and CKD-related targets. Furthermore, network visualization was accomplished by using Cytoscape software (version 3.6.1) and the MCODE plug-in in Cytoscape was used to identify the hub genes. Next, Gene Ontology (GO) and the Kyoto Encyclopedia of Genes and Genomes (KEGG) functional enrichment were performed by using the clusterProfiler software package of the R platform.

### 2.4. Evaluation of Active Ingredients and Hub Genes' Interaction by Molecular Docking

The crystal structures of hub genes were obtained from RCSB PDB database (https://www.rcsb.org/pdb/) and Pymol2 software was used to remove solvents, and organic and AutoDock 4.2.6 software was employed to add polar hydrogen atoms and charges to the protein crystal structures. Additionally, the structures of the active ingredients were retrieved from NCBI PubChem, and AutoDock Vina software was used to perform the interaction between the targets and the active ingredients. What's more, PLIP was employed to expand the scope of the protein-ligand interaction profiler [21].

### 2.5. UUO-Induced Animal Model and A&P Treatment

The male C57BL/6 mice (aged at 8 weeks, weighing about 20 g) were obtained from Dashuo BioTechnique Co. Ltd. (Chengdu, China) and raised in an environment with a constant temperature of 20–22°C and a humidity of 50%–60%. The mice were randomly divided into Sham group, UUO group, A&P low group (1972 mg/kg/day, added to the experimental diets) and A&P high group (7888 mg/kg/day, added to the experimental diets) (*n* = 8 per group). All mice were sacrificed on the 7th day. All animal handling and experimental procedures were approved by the Animal Ethics Committee of Southwest Medical University.

### 2.6. Histology and Immunohistochemical Staining

Mouse kidney tissues were carefully isolated, fixed with 4% paraformaldehyde, and embedded in paraffin. Then, the paraffin-embedded specimens were sectioned, deparaffinized, and rehydrated with gradient ethanol and subjected to subsequent detections including HE, Masson trichrome staining, and immunohistochemistry. Immunohistochemistry was performed according to the following procedures: the sections were immersed in citric acid antigen repair buffer based on microwave-based antigen retrieval technology as described before [7]. Then, the sections were reacted with indicated primary antibodies including *α*-SMA (1 : 100, China, Boster), fibronectin (1 : 100, USA, Abcam) overnight at 4°C. Immediately after that, the sections were rinsed with PBS, incubating with Biotin-Streptavidin HRP-based SPlink Detection Kits (ZSGB-Bio, China), and color was developed with DAB. Finally, Counterstaining was performed with hematoxylin. Signals were detected with a Virtual Slide Microscope (VS120, Olympus, Japan).

### 2.7. Western Blot Assay

Total proteins of the kidney cortex were extracted RIPA lysis buffer (Beyotime, China), and the concentration was measured by the BCA method. The samples were transferred to PVDF membrane, blocked with 5% BSA at room temperature for 1 hour, and the PVDF membrane was incubated with the corresponding antibodies including anti-HSF1 (1 : 500, Santa cruz (cat# sc-8402), USA)/RELA (1 : 500, Santa cruz (cat# sc-17757), USA) at 4°C overnight. Furthermore, the membranes were incubated with the corresponding HRP-conjugated secondary antibody after rinsing and exposed with ECL Chemiluminescence Kit (Thermo, Waltham, MA, USA). Image J software was conducted to analyze the gray value, and the protein levels were corrected for the housekeeping gene GAPDH.

### 2.8. RNA Isolation and Real-Time PCR

Total RNA was isolated by using Trizol (Invitrogen, cat# 15596018) according to the manufacturer's instructions. Next, a reverse transcription kit (Vazyme, cat# R323-01) was used to conduct reverse transcription. Then, a quantitative real-time PCR was performed to detect the expression of genes at the transcriptional level using a LightCycler® 480 II Real-Time PCR System (Roche, Germany). The sequence of primers used is listed in Table 2, and the relative expression of genes was normalized against GAPDH by the 2−ΔCt method.

### 2.9. Statistical Analysis

All the results are expressed as the mean ± standard deviation (SD). Statistical analyses were performed with one-way ANOVA and the Newman-Keuls multiple comparison tests by GraphPad Prism 7.0 (GraphPad Software, La Jolla, CA, USA). *P*-values <0.05 were considered to be statistical significance.

## 3. Results

### 3.1. Identifying the Potential Active Ingredients and Regulatory Proteins of A&P

**Figure 2:**
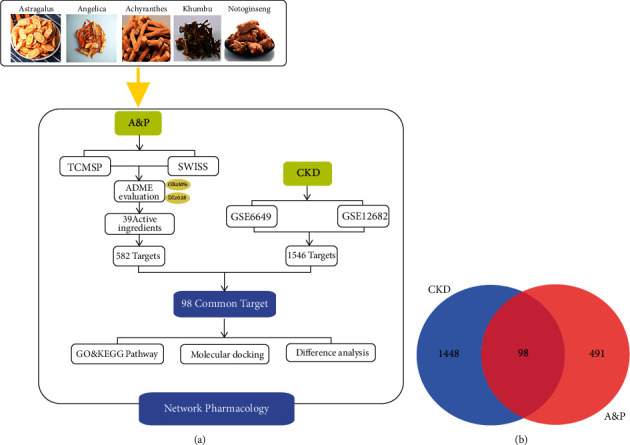
Schematic diagram of network pharmacology analysis. (a) Network Pharmacology. (b) Venn diagram of drug-disease common targets.

To screen the active components of A&P, we preliminarily obtain the main ingredients of A&P from the TCMSP platform. Furthermore, a total of 37 candidate components of A&P was filtered according to oral availability (OB) ≥ 30% and drug likeness (DL) ≥ 0.18, of which 15 active ingredients in “huangqi”, 1 active ingredients in “danggui”, 11 active ingredients in “niuxi”, 4 active ingredients in “sanqi”, and 6 active ingredients in “kunbu.” Detailed information on these active compounds is listed in Table 3. The protein targets of the 37 active compounds were then obtained from the intersection of the Swiss Target Prediction database and the TCMSP platform. Following the removal of duplicates, 1157 potential targets of the 37 compounds in total were employed for further analysis (as listed in Figure 2(a)).

### 3.2. Construction of the Integrated Network of A&P on CKD

In order to obtain CKD related-targets, we retrieved the NCBI GEO database with “CKD” as a keyword (conditional parameters: gene expression profile microarray data, the number of samples in a single group is greater than 20);a total of 2 sets of microarrays are obtained, named “GSE6649″ and “GSE12682”. After removing the redundant genes, we obtained 1546 CKD-related target genes by intergating the two microarrays (The differential genes are listed in Supplementary Materials S1). On the above basis, an integrated network of A&P on CKD was constructed, and a Venn diagram on the potential protein targets of A&P and CKD related targets was drawn including a total of 98 genes (as shown in Figure 2(b)).

### 3.3. Enrichment Analysis by GO and KEGG Analyses

**Figure 3:**
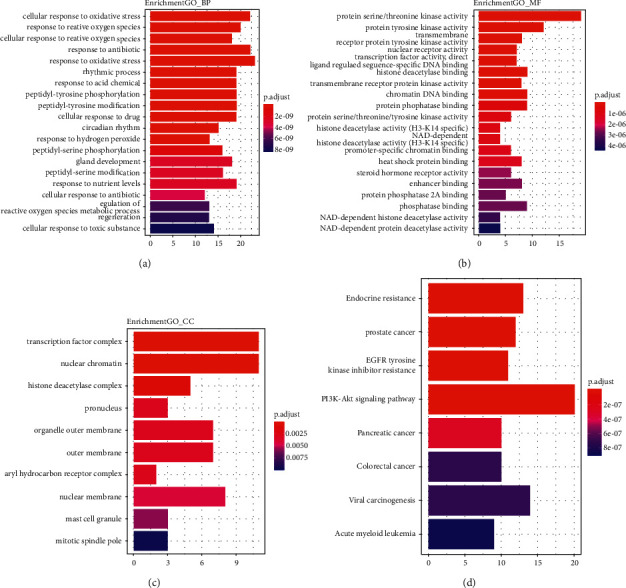
GO and KEGG enrichment of drug-disease common targets. (a) Biological process. (b) Molecular function. (c) Cellular component. (d) KEGG enrichment analysis.

To further elaborate on the potential biological function of A&P on CKD, we performed GO and KEGG analysis. The results showed that the biological processes involved in the coacting targets are closely related to factors such as cell oxidative stress and protein serine and threonine kinase activity (Figures 3(a) and 3(b)). In addition, these molecules are also mainly involved in transcription regulation (Figure 3(c)). KEGG analysis results revealed that A&P therapeutic targets for CKD are significantly enriched in the AKT signaling pathways (Figure 3(d)).

### 3.4. Selection of Hub Genes of A&P on CKD

**Figure 4:**
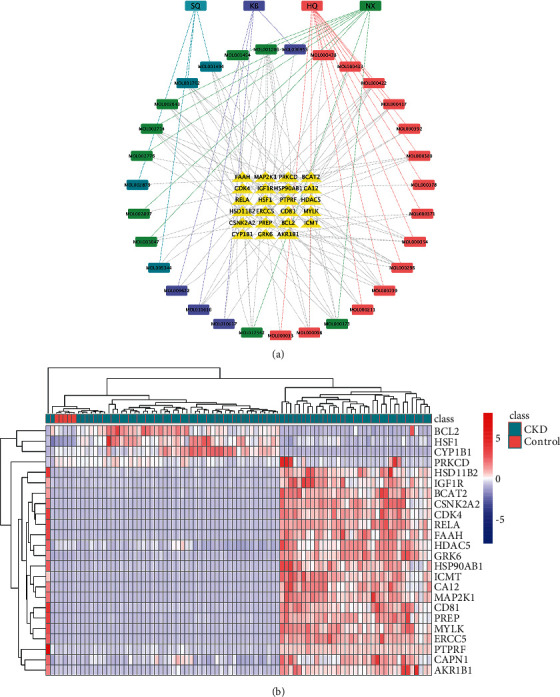
Analysis of network pharmacology and differential analysis. (a) Regulatory network of TCM compound prescription (note: blue represents the potential active component of *Panax notoginseng*; Purple represents Cambrian; red represents *Astragalus membranaceus*; green stands for *Achyranthes bidentata*; yellow represents the core gene.). (b) Differential gene analysis of disease-targets by microarrays.

To gain further insights into the relationship between the potential active ingredients and coaction genes, a network of Chinese herbal compound was built by using cytoscape software (Figure 4(a)). In order to obtain more accurate targets for A&P intervention in CKD, we tightened up the selection criteria, and HSF1 and RELA exhibited to be the central hub of the integrated network. What's more, increasing evidence also revealed that HSF1 and RELA play a vital role in fibrosis and inflammation related disease [22, 23]. Based on above, we regarded HSF1 and RELA as hub genes.

### 3.5. The Protective Effect of A&P on CKD

**Figure 5:**
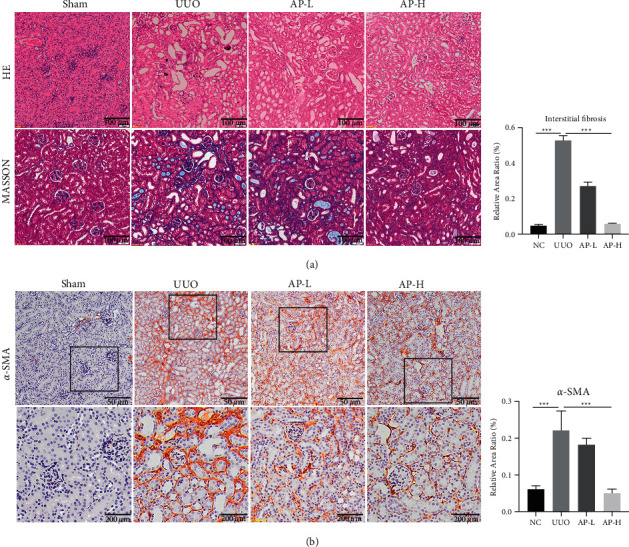
Effects of AP on renal injuries in UUO kidneys. (a) HE staining and Masson staining of mouse kidneys (scale bar = 100 *μ*m). (b) Immunohistochemical staining of *α*-SMA in mouse kidneys (scale bar = 50 *μ*m). ^*∗∗∗*^*P* < 0.001 with the indicated group.

Next, we determined the effect of A&P on CKD in the rodent model. Firstly, the pathological results from histology discovered that increased renal tubulointerstitial fibrosis, tubular dilation, glomerular sclerosis, and flattened tubular epithelial cells in the UUO group while the lesion was significantly modified after AP treatment in a dose-dependent manner (Figure 5(a)). In addition, the fibrosis lesions in the UUO group showed a similar tendency as evidenced by the Masson staining and the immunohistochemistry of *α*-SMA (Figures 5(a) and 5(b)). In conclusion, A&P administration could dramatically improve the kidney injury of CKD.

### 3.6. Verification of Hub Genes and Docking Analysis

**Figure 6:**
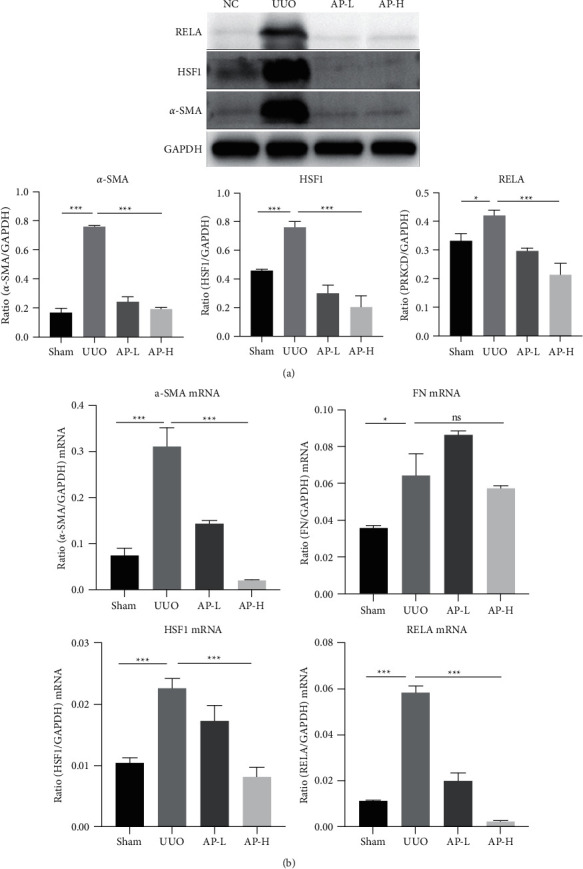
Hub genes' verification. (a) Effect of AP on the protein level of HSF1, and RELA was detected by western blotting analysis. (b) Effect of AP on mRNA expression of *α*-SMA, FN, HSF1, and RELA according to real-time PCR analysis. ^*∗*^*P* < 0.05 and ^*∗∗∗*^*P* < 0.001 with the indicated group.

**Figure 7:**
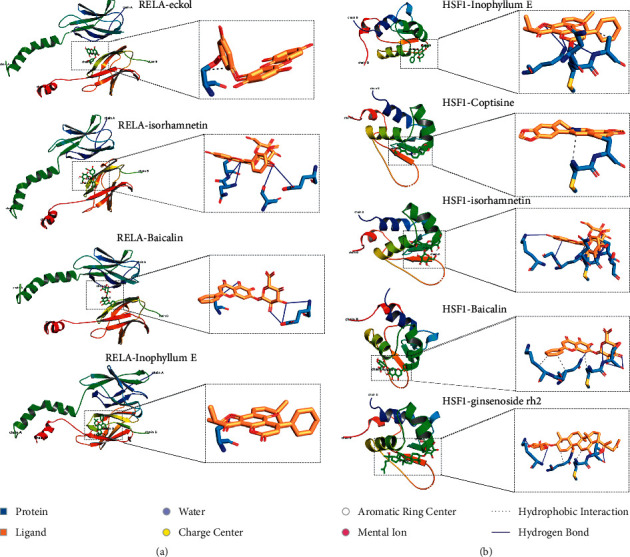
Molecular docking of some potential active ingredients and hub genes. (a) The interaction of RELA and indicated active ingredients. (b) The interaction of HSF1 and indicated active ingredients.

We next assessed the expression of hub genes including HSF1 and RELA after A&P on CKD. As shown in Figures 6(a) and 6(b), the expression of HSF1 and RELA both at the transcriptional and protein levels in UUO was markedly increased, whereas A&P administration reversed the aberrant upregulation (Figures 6(a) and 6(b)). To gain better elaboration of A&P on the effect of HSF1 and RELA, we performed molecular docking between active ingredients of A&P and hub genes. The binding affinity between active ingredients and hub genes is listed in Table 4 and visualized in Figures 7(a) and 7(b). It should be noted that active ngredients including Inophyllum E, Baicalin displayed relatively stable binding free energy with both RELA and HSF1. In summary, the active ingredients of A&P on RELA and HSF1 may partially explain the treatment effect of A&P on CKD.

## 4. Discussion

It is well accepted that the global incidence of CKD is extremely high year after year. Currently, there is no specific treatment for CKD, and interventions have limited efficacy [24]. Therefore, there is an urgent need to search for effective and safer treatment. Herbal medicine is the main treatment of TCM, and A&P has been used in the treatment of CKD patients for a long time [25]. Our recent research has revealed that A&P can improve renal injury in diabetic nephropathy partly through inhibiting apoptosis or promoting autophagy [25]. However, up to now, evidence on A&P improving CKD remains to be poorly elaborated. In light of this, an integrated network was constructed to scientifically elaborate the potential mechanism of A&P on CKD in this paper.

First, we retrieved the potential active ingredients and regulatory proteins of A&P from the TCMSP and Swiss Target Prediction database and 37 candidate components and corresponding potential targets. Secondly, an integrated network of A&P on CKD was constructed, and hub genes including HSF1 and RELA were identified. Immediately after that, we validated the reliability of hub genes in UUO mice in the rodent model. Consistent with the expectation, A&P treatment reversed the aberrant upregulation of HSF1 and RELA in UUO in a dose dependent manner both at the transcriptional and protein levels. What's more, the results of molecular docking also suggest that potential effective compounds have relatively stable binding energy with HSF1 and RELA, which provides a strong theoretical basis for us to further explore the role of HSF1 and RELA in A&P delaying or even reversing CKD induced by renal fibrosis.

Renal fibrosis in the process of CKD is the proliferation of stromal cells such as fibroblasts. Previous studies suggest that normal renal homeostasis requires HSF1 dependent selective HSPs transcription to protect renal cells from oxidative stress under physiological conditions, and HSP27 activation may have great potential in the treatment of renal IRI. In addition, HSF1 is also involved in the process of liver fibrosis, and the mechanism may be that mir-455-3p reduces the activation of hepatic stellate cells and liver fibrosis by inhibiting the expression of HSF1 [23]. In the current study, we observed that HSF1 laid a central role in A&P on CKD and multiple active ingredients exhibited an intense binding free energy with HSF1. RELA, also known as p65, is one of NF-*κ*B, a dimeric monopeptide protein that is ubiquitous in mammals and has a wide range of biological functions. It is well accepted that RELA participates in inflammation and oxidative stress [26]. Similar to HSF1, active ingredients of A&P such as eckol docked well into the RELA binding cavity with strong binding affinity. In conclusion, molecular docking and network pharmacology were used for the first time to clarify the material basis of A&P in the treatment of CKD. This study is expected to broaden the selection range of AP treatment methods and further prove the feasibility of network pharmacology in the analysis of traditional Chinese medicine prescriptions.

It should be remarked here that there were also shortcomings to the study: firstly, the active ingredients of A&P were obtained from the TCMSP platform. This may neglect some potential active ingredients. In our future study, we aim to resolve the active ingredients of A&P by HPLC-MS. Secondly, it is better to perform RNA sequencing to elucidate the targets of A&P rather than integrating the data from microarrays, which causes the loss of information, especially in the epigenetic field. Hence, we plan to carry out RNA sequencing to obtain unbiased targets. In total, our current study laid the foundation for elucidating the protective effect of A&P.

## Figures and Tables

**Table 1:** The component of A&P.

Components	Content (gram)
*Astragalus propinquus* Schischkin	3
*Ecklonia kurome*	3
*Angelica sinensis*	3
*Achyranthes bidentata*	3
*Panax notoginseng*	1

**Table 2:** Primers used for RT-PCR analysis.

Gene name	Gene sequence
GAPDH	Forward primer: 5'-AGGTCGGTGTGAACGGATTTG-3'
	Reverse primer: 5'-TGTAGACCATGTAGTTGAGGTCA-3'

HSF1	Forward primer: 5'-AACGTCCCGGCCTTCCTAA-3'
	Reverse primer: 5'-AGATGAGCGCGTCTGTGTC-3'

RELA	Forward primer: 5'-AGGCTTCTGGGCCTTATGTG-3'
	Reverse primer: 5'-TGCTTCTCTCGCCAGGAATAC-3'

**Table 3:** Basic information of AP's potential ingredients.

Drug	Molecule name	MolID	OB	DL
Huangqi	Mairin	MOL000211	55.38	0.78
Huangqi	Jaranol	MOL000239	50.82	0.29
Huangqi	Hederagenin	MOL000296	36.91	0.75
Huangqi	(3S,8S,9S,10 R,13R,14S,17R)-10,13-dimethyl-17-[(2R,5S)-5-propan-2-yloctan-2-yl]-2,3,4,7,8,9,11,12,14,15,16,17-dodecahydro-1H-cyclopenta[a]phenanthren-3-ol	MOL000033	36.23	0.78
Huangqi	Isorhamnetin	MOL000354	49.6	0.31
Huangqi	3,9-Di-O-methylnissolin	MOL000371	53.74	0.48
Huangqi	7-O-Methylisomucronulatol	MOL000378	74.69	0.3
Huangqi	(6aR,11aR)-9,10-dimethoxy-6a,11a-dihydro-6H-benzofurano[3,2-c]chromen-3-ol	MOL000380	64.26	0.42
Huangqi	Bifendate	MOL000387	31.1	0.67
Huangqi	Formononetin	MOL000392	69.67	0.21
Huangqi	Calycosin	MOL000417	47.75	0.24
Huangqi	Kaempferol	MOL000422	41.88	0.24
Huangqi	FA	MOL000433	68.96	0.71
Huangqi	(3R)-3-(2-Hydroxy-3,4-dimethoxyphenyl)chroman-7-ol	MOL000438	67.67	0.26
Huangqi	Quercetin	MOL000098	46.43	0.28
Niuxi	Poriferasta-7,22E-dien-3beta-ol	MOL001006	42.98	0.76
Niuxi	*β*-Ecdysterone	MOL012542	44.23	0.82
Niuxi	Berberine	MOL001454	36.86	0.78
Niuxi	Coptisine	MOL001458	30.67	0.86
Niuxi	Wogonin	MOL000173	30.68	0.23
Niuxi	Delta 7-stigmastenol	MOL002643	37.42	0.75
Niuxi	Baicalein	MOL002714	33.52	0.21
Niuxi	Baicalin	MOL002776	40.12	0.75
Niuxi	Epiberberine	MOL002897	43.09	0.78
Niuxi	Beta-sitosterol	MOL000358	36.91	0.75
Niuxi	Inophyllum E	MOL003847	38.81	0.85
Kunbu	CLR	MOL000953	37.87	0.68
Kunbu	Saringosterol	MOL010615	43.48	0.62
Kunbu	24-Methylenecholesterol	MOL010625	43.54	0.76
Kunbu	Fucosterol	MOL009622	43.78	0.76
Kunbu	1553-41-9	MOL010617	45.66	0.21
Kunbu	Eckol	MOL010616	87.06	0.63
Danggui	Stigmasterol	MOL000449	43.83	0.76
Sanqi	Mandenol	MOL001494	42	0.19
Sanqi	DFV	MOL001792	32.76	0.18
Sanqi	Diop	MOL002879	43.59	0.39
Sanqi	Ginsenoside rh2	MOL005344	36.32	0.56

**Table 4:** Molecular docking of A&P's potential ingredients and core genes.

Drug	Molecule name	MolID	Affinity (kcal/mol)	
			HSF1	RELA
Niuxi	Inophyllum E	MOL003847	−7.1	−7.3
Niuxi	Coptisine	MOL001458	−7.0	−7.4
Huangqi	Isorhamnetin	MOL000354	−6.8	−7.5
Niuxi	Baicalin	MOL002776	−6.8	−7.4
Sanqi	Ginsenoside rh2	MOL005344	−6.7	−6.7
Niuxi	Berberine	MOL001454	−6.6	−6.6
Huangqi	Hederagenin	MOL000296	−6.4	−7.1
Niuxi	Ecdysterone	MOL012542	−6.4	−6.7
Niuxi	Wogonin	MOL000173	−6.3	−6.5
Niuxi	Baicalein	MOL002714	−6.3	−6.6
Kunbu	Eckol	MOL010616	−6.3	−7.6
Huangqi	Jaranol	MOL000239	−6.2	−6.6
Niuxi	Epiberberine	MOL002897	−6.2	−6.2
Danggui	Stigmasterol	MOL000449	−6.2	−7.1
Huangqi	Quercetin	MOL000098	−6.1	−6.7
Niuxi	Poriferasta	MOL001006	−6.1	−6.6
Huangqi	Mairin	MOL000211	−6.0	−7.2
Kunbu	Fucosterol	MOL009622	−6.0	−6.9
Huangqi	7-O-Methylisomucronulatol	MOL000378	−5.9	−6.6
Huangqi	Formononetin	MOL000392	−5.9	−6.6
Huangqi	Calycosin	MOL000417	−5.9	−6.7
Niuxi	Beta-sitosterol	MOL000358	−5.9	−6.8
Kunbu	24-Methylenecholesterol	MOL010625	−5.9	−7.0
Sanqi	Diop	MOL002879	−5.9	−6.9
Kunbu	Saringosterol	MOL010615	−5.7	−6.9
Sanqi	DFV	MOL001792	−5.7	−6.0
Huangqi	Bifendate	MOL000387	−5.5	−6.0
Huangqi	FA	MOL000433	−5.4	−5.3
Huangqi	Kaempferol	MOL000422	−5.3	−6.8
Sanqi	Mandenol	MOL001494	−4.9	−4.5
Kunbu	1553-41-9	MOL010617	−4.0	−4.1

## Data Availability

The data used to support the findings of this study are included within the article.

## Supplementary Materials

Supplementary Materials The supplementary materials contain 1 file, the potential targets of A&P.
