# Supplementary material for: Exploring the Mechanism of Astragalus propinquus Schischkin and Panax Notoginseng (A&P) Compounds in the Treatment of Renal Fibrosis and Chronic Kidney Disease Based on Integrated Network Analysis
Source: Evid Based Complement Alternat Med. 2022 Feb 27;2022:2646022. doi: 10.1155/2022/2646022 (PMC8898808; doi:10.1155/2022/2646022)
Supplement: Supplementary Materials — The supplementary materials contain 1 file, the potential targets of A&P. [file 2646022.f1.pdf]

Supplement1 . Information of differenial genes

Gene Names	logFC	AveExpr	t	P.Value	adj.P.Val	B
MYH11	-2.629446089	3.65453355	-3.475980491	0.000787409	0.01832181	-0.674034872
PXN	-2.268594415	7.334018413	-3.43436084	0.000902156	0.019390296	-0.795331428
CNN1	-2.052070865	3.218705975	-3.302208108	0.001379786	0.021766834	-1.173108548
SEMA3B	-2.012891669	5.411557127	-1.9142515	0.05878484	0.095363445	-4.383159791
RBM5	-1.667310049	4.449064018	-2.508060708	0.013942979	0.046169035	-3.189458201
AQP3	-1.585493083	3.163043632	-3.212688316	0.001828582	0.025017592	-1.422512658
PRPF31	-1.218741197	3.76236363	-1.854959849	0.066896113	0.105629763	-4.486376717
PDAP1	-1.189145402	2.848702957	-2.29090327	0.02431881	0.057564126	-3.659010571
AKT2	-1.093650805	2.681728427	-3.281326111	0.001474137	0.022897772	-1.231761335
NUDCD3	-0.86708074	2.46775163	-3.151491576	0.002210269	0.026423162	-1.589920829
PRKAB1	-0.776901788	2.640041853	-2.637296405	0.009854199	0.040162565	-2.892646147
MZF1	-0.771049706	2.665679855	-3.428355428	0.00091996	0.019482983	-0.81274277
MARK2	-0.623893243	2.799376649	-1.786030132	0.077482986	0.116299705	-4.602601237
RBM14	0.550269692	4.369705682	2.105430473	0.038056853	0.072726693	-4.030208461
SMG7	0.569278281	2.246275057	2.046671437	0.043625179	0.078790334	-4.141932192
LMO7	0.572170937	2.679415412	2.16859882	0.032766732	0.067184838	-3.90692895
RNASEH2A	0.574346581	3.164563122	1.8447176	0.068388143	0.107438167	-4.503903702
NR3C1	0.585797199	4.189644772	1.816017334	0.072717847	0.111200641	-4.552539205
PKNOX1	0.598748536	2.447211588	3.063166398	0.002893462	0.029105605	-1.827041318
ZNF24	0.6411048	6.633214615	2.137675188	0.035270701	0.068936914	-3.967688408
SUPT4H1	0.657234059	7.24131921	2.1506435	0.034201084	0.068269745	-3.942303225
PSMD3	0.666069919	5.793773107	2.3171298	0.022780009	0.055901418	-3.604280282
TPD52	0.670356352	4.930585209	2.047915635	0.043500383	0.078790334	-4.139596134
ACBD3	0.679175574	3.951439336	1.770718899	0.080014084	0.119173192	-4.627864315
BLMH	0.695929099	5.469606396	1.8617446	0.065922831	0.104315964	-4.47471708
TMEM11	0.73819503	4.827285211	1.973360334	0.051541258	0.088047275	-4.277299766
COPE	0.764128847	4.621814447	1.918900798	0.058185492	0.094789011	-4.374940039
SP100	0.767506793	7.431235479	2.332501453	0.021918648	0.055099562	-3.571947183
RPS7	0.777324473	9.950032743	2.413368903	0.017845106	0.050069936	-3.398764617
DVL1	0.783527333	7.511649404	2.856855181	0.005320159	0.035007172	-2.359666022
TIMM17A	0.78388901	6.168941006	3.157639345	0.00216881	0.026401425	-1.573217663
KIAA0391	0.788156282	2.339634683	3.336670429	0.001236359	0.021180266	-1.07568549
CTNNBIP1	0.792867866	6.270298736	2.098498697	0.038680063	0.073532719	-4.043536921
HSF1	0.813875599	7.040897402	3.25596891	0.001596833	0.022969498	-1.302596593
ZNF318	0.822021707	1.919126574	2.51751946	0.013598714	0.045756728	-3.168168389
POLR2L	0.831795323	4.294621619	2.739529983	0.007426298	0.038060621	-2.648907969
QSOX1	0.836233776	6.214013411	2.699122178	0.008312009	0.038651569	-2.746178318
PLOD2	0.839906186	2.634766137	2.584844991	0.011361001	0.042835027	-3.014645933

TUSC2	0.840171142	6.608346466	3.382269028	0.001068022	0.019893518	-0.945591495
FAM120A	0.863161571	4.305585947	2.684808863	0.008648111	0.039313143	-2.78034197
RASSF2	0.863676067	2.239021261	1.787552852	0.077234932	0.116040043	-4.60007776
UCHL1	0.874083658	6.795799669	1.95683207	0.053485939	0.089940802	-4.307197237
SF3B3	0.895230381	3.70849733	1.874584637	0.06411338	0.10218483	-4.452544058
TIMP2	0.902301148	4.281427225	2.07591962	0.040771577	0.075851814	-4.086677132
CPT1A	0.903903896	1.863718967	2.832617947	0.005704041	0.035220999	-2.420241783
PICALM	0.904178384	2.627927781	3.473430347	0.000794024	0.01832181	-0.681498564
RBMX2	0.905744849	3.052131319	2.300625437	0.023738004	0.057004657	-3.638786189
ANP32A	0.908941008	3.438295861	3.661827926	0.000423519	0.014498194	-0.119222215
TSN	0.924442564	3.575848936	3.389742055	0.00104258	0.019697314	-0.924142427
RPA1	0.92939359	5.245473312	2.650106051	0.009514855	0.039795714	-2.862535328
RPL35	0.934473356	9.631358296	1.689523138	0.094601977	0.135295704	-4.758448192
STT3A	0.93934809	4.270254141	3.024950825	0.003245946	0.029825904	-1.927965896
RNGTT	0.941092521	2.137076181	3.062503112	0.002899265	0.029105605	-1.828801667
SMARCD3	0.944121017	5.069089376	2.486105145	0.014772415	0.047186269	-3.238609422
NCBP2	0.945193434	3.30443042	2.658394559	0.009300965	0.03968189	-2.842986233
APRT	0.946225023	7.482021231	2.313944099	0.022962218	0.055904864	-3.610957607
STX5	0.953934122	3.634492913	3.265433572	0.001549958	0.022969498	-1.276206921
DUSP4	0.954690057	1.654179431	2.260346951	0.026226586	0.05989114	-3.722081551
FCER1G	0.955182606	2.899890882	2.730502587	0.007616354	0.038187781	-2.670744031
YWHAH	0.965828096	3.166064698	2.547958807	0.012542053	0.044579376	-3.099187808
MECP2	0.967465146	4.553142896	4.057482979	0.000105893	0.008474605	1.129959906
ATP6V1A	0.972120895	3.482404377	2.672940544	0.008936116	0.039389826	-2.808553626
SUMO3	0.974628	4.068467351	2.219633158	0.028972217	0.06278676	-3.804950438
MAN2A2	1.012999632	5.015221987	1.832826701	0.070155129	0.108895412	-4.524139536
MTA2	1.014735426	2.14454169	4.484267283	2.16E-05	0.003333639	2.572071724
CD82	1.025412732	4.347344037	2.255207873	0.02656005	0.060207972	-3.732615365
HSPB1	1.029440364	8.175816819	1.718910659	0.089089719	0.129084073	-4.711845253
AMFR	1.036211038	3.92674775	1.805979323	0.074285042	0.113041053	-4.5693831
PYGB	1.039067241	5.028418177	2.590521859	0.011188428	0.042499534	-3.001542552
RARA	1.039187736	2.432688499	3.114462884	0.002476009	0.027940948	-1.689980983
DHX8	1.049615274	3.17262708	1.95348283	0.053887505	0.090260112	-4.313227585
TRAM1	1.052944273	7.178775049	3.435293835	0.000899419	0.019390296	-0.792624355
FKBP2	1.069820685	8.668400682	3.404851283	0.000992873	0.019697314	-0.880666146
TAF15	1.07387539	6.874860545	1.840175781	0.069058616	0.10787954	-4.511647195
GOLGA1	1.082333074	4.086182974	2.745652936	0.007299862	0.038060621	-2.634063028
BAZ2A	1.082394996	1.937751194	2.294217053	0.024119451	0.057367187	-3.652125623
SPTBN1	1.089711437	2.031153202	2.406280929	0.018173057	0.050531558	-3.414150159
IQCE	1.101593944	4.753608151	3.565492381	0.000585591	0.016166481	-0.409472956

CXCL12	1.122378895	6.412156341	1.827788159	0.070915264	0.109483141	-4.532677615
USO1	1.130167493	5.533261916	2.466143474	0.015564614	0.047973225	-3.282972734
TMEM184B	1.131171377	6.822500802	3.973395918	0.000143201	0.009224523	0.857066098
CTSF	1.137292179	8.14809448	2.943328431	0.004135836	0.032292944	-2.140096414
PDLIM5	1.141509827	2.642571157	3.323382061	0.00128992	0.021228506	-1.11334305
DRG2	1.15111079	4.227216374	1.940821261	0.055428798	0.091846647	-4.335939524
XRCC1	1.152624964	5.415491578	2.813830927	0.006018897	0.035927472	-2.46690225
NFKBIA	1.156054939	7.955601691	2.469870167	0.015413892	0.047973225	-3.274713937
PLD1	1.156417918	4.402508221	1.734082584	0.08634854	0.125583108	-4.687492085
PSMB10	1.159535321	5.556179083	2.204398759	0.030062501	0.063685405	-3.8356139
DLGAP4	1.172618702	1.082490617	2.230060336	0.02824607	0.061992799	-3.783854481
PMVK	1.192678478	6.609498116	2.314324221	0.022940409	0.055904864	-3.610161284
SV2A	1.206989062	1.441321202	2.992625138	0.003574731	0.03094564	-2.012539235
AKAP11	1.210466435	5.760677483	2.075040845	0.040854912	0.075915497	-4.088347624
VARS	1.211548307	3.354283802	1.702335581	0.092165465	0.1325526	-4.738222416
ADARB1	1.216181427	3.573173448	1.764529034	0.081056542	0.12037792	-4.638020064
KIAA0100	1.21667375	6.473892256	1.834472167	0.069908365	0.108730717	-4.521346501
MGP	1.219957006	6.917227192	1.679886817	0.096468869	0.137330453	-4.773565975
DARS	1.230361149	6.739975457	3.650051369	0.00044076	0.014498194	-0.155006585
RANBP3	1.231713755	5.268549962	5.121721923	1.73E-06	0.000533736	4.88367622
STX4	1.238043063	5.535037187	2.785766502	0.006518969	0.037327133	-2.536124657
DNPEP	1.238121955	5.452527184	1.888249763	0.062233643	0.100013735	-4.428792156
AKR7A2	1.239173773	8.269230738	1.840839385	0.068960312	0.10787954	-4.510516894
SAR1A	1.239481717	5.943910503	3.584000183	0.000550482	0.015760107	-0.354150704
LSP1	1.243978232	1.604874059	2.086023324	0.039823909	0.074537244	-4.067424779
ACADVL	1.255265374	9.324171496	1.933641127	0.056319319	0.092824805	-4.348758981
OS9	1.257890352	6.919608238	1.801037511	0.075066839	0.113777778	-4.577643754
HTT	1.259120525	3.669629858	2.120660726	0.036717986	0.071046315	-4.000784656
LMNA	1.263320968	7.091798613	2.931099574	0.004287081	0.032458075	-2.171472333
ZNF593	1.267291883	5.371649671	2.648762569	0.009549942	0.039795714	-2.865699169
VPS41	1.270832478	2.35755732	4.150583099	7.55E-05	0.00648311	1.436561683
HSPBP1	1.27289388	4.432461111	1.838565557	0.069297636	0.10799813	-4.514388285
HSD11B2	1.286174776	8.795758026	1.772151238	0.079774443	0.119054659	-4.625509562
CUX1	1.301587286	5.464352189	1.673197241	0.097782416	0.138308888	-4.784013217
RPL18	1.303774273	9.154932385	1.804547687	0.074510829	0.11304587	-4.571778357
SSB	1.304795905	7.095063714	4.032774453	0.000115759	0.008474605	1.049370654
RHOC	1.313724338	7.836540272	2.935550099	0.004231457	0.032458075	-2.160065819
MAP3K3	1.320455723	5.91732317	3.545794715	0.000625279	0.016310363	-0.468119648
KIF2A	1.321953506	2.466563695	6.305996047	1.06E-08	1.64E-05	9.561898046
BYSL	1.331197887	3.180867147	2.431445024	0.017032497	0.049027922	-3.359349215

DGAT1	1.332408723	4.409602741	1.698910994	0.092811638	0.133228219	-4.743642467
BAG1	1.334866293	9.046181828	2.297515378	0.023922455	0.057162465	-3.645264065
POLR2D	1.343434876	1.955264761	2.421645436	0.017468844	0.04928254	-3.380749225
ITM2A	1.343776534	4.33677452	1.78944926	0.076926923	0.115689711	-4.596932212
ARHGEF12	1.348723065	4.578450043	3.861007082	0.000213079	0.01098065	0.498454202
SNRPD1	1.357143141	6.437932156	3.639213414	0.000457214	0.014726098	-0.187864894
AXL	1.357740253	3.50549844	2.052058996	0.043087004	0.078496147	-4.131807446
CEP104	1.358995954	5.02124687	3.724553913	0.000341965	0.013912586	0.072778618
SEPW1	1.375351352	8.512623662	2.967933122	0.003846338	0.032013404	-2.076645639
RRBP1	1.382022056	5.05952067	1.961910014	0.052881953	0.089725414	-4.298036337
PPRC1	1.387584319	3.626995601	2.720624802	0.007829385	0.038242915	-2.694567844
GYS1	1.388176313	4.682169606	1.709433561	0.090837874	0.131247994	-4.726956079
ATP6V0B	1.388240684	7.512115008	1.748164698	0.083866615	0.123014979	-4.664709847
CDK2AP2	1.390383356	3.961071931	2.228819645	0.028331627	0.062040645	-3.786369219
AKAP8	1.398263703	4.355016167	2.164711014	0.033072786	0.067466138	-3.914610768
TOE1	1.399845199	4.363567056	2.206987496	0.029874741	0.063685405	-3.83041662
TGFBI	1.409375225	6.135265246	1.685767099	0.095326122	0.135954045	-4.764350411
TYROBP	1.410267935	5.629670533	1.945561735	0.05484742	0.091471533	-4.327452025
LAMP1	1.423513286	10.61049389	3.656051901	0.000431894	0.014498194	-0.136783756
PDCD6	1.428439853	7.243346544	2.828581289	0.00577039	0.035220999	-2.430289119
FH	1.430693956	6.379213695	1.821061559	0.071940758	0.110447281	-4.544042326
CLTA	1.43337115	7.944836715	1.97878487	0.050916275	0.08765764	-4.267437478
HNRNPM	1.433616045	8.036465484	3.901245716	0.000184966	0.010542713	0.626030302
RPS16	1.44034741	9.872229149	1.734550254	0.086265157	0.125579975	-4.686738238
CD81	1.441914688	8.673339313	1.84289594	0.068656404	0.107540831	-4.507011625
SNRPF	1.442672305	6.536849122	3.25119564	0.001620973	0.022991042	-1.315883006
TIMM17B	1.44534279	3.818413936	1.876636173	0.063828171	0.101835245	-4.448988329
MFN2	1.461881572	7.543025471	3.786974244	0.000275785	0.012181799	0.266158109
FAM50A	1.46607086	5.650564289	2.059898021	0.042314143	0.077693189	-4.117032666
IGFBP7	1.46950254	11.10329189	2.869049235	0.005136128	0.03496315	-2.329028793
TESK1	1.476959774	6.40243914	3.220831196	0.001782711	0.024816187	-1.400047067
CAV1	1.494627563	3.646255553	2.471136722	0.015362966	0.047973225	-3.271904642
PVRL2	1.496441876	5.813092334	4.579184319	1.50E-05	0.002579887	2.904982599
ATG9A	1.500802686	4.45737485	1.863574564	0.065662362	0.104067316	-4.471565557
GRK6	1.502465159	2.584094373	2.227987254	0.028389156	0.062078691	-3.788055684
EZH2	1.51170035	2.13230835	2.917626523	0.004459607	0.032725083	-2.20591712
EEF1D	1.515014547	9.27848555	1.922891321	0.057675193	0.094455347	-4.367870389
TPST1	1.520751919	5.074181206	3.083981067	0.002716759	0.028788954	-1.771644875
C17orf75	1.523621801	2.626006822	1.863321126	0.065698383	0.104067316	-4.472002193
PDHA1	1.526947607	7.822796725	3.041433579	0.003089299	0.029744246	-1.884560818

MON1B	1.527850345	1.989047154	2.155639577	0.033796596	0.067849344	-3.932486777
PUM1	1.527998248	6.584971571	3.392072751	0.001034763	0.019697314	-0.917445509
NFYA	1.5375673	1.917762599	5.870945402	7.25E-08	3.74E-05	7.795750889
EIF5B	1.541068655	2.56420973	3.669279126	0.000412943	0.014498194	-0.096537764
CCDC86	1.543681971	5.436786982	4.021390008	0.000120596	0.008474605	1.012350951
CD93	1.547458347	0.681665136	2.354713588	0.020724888	0.054031496	-3.524893773
PLD3	1.550263093	3.473933821	1.677580253	0.096920149	0.137592792	-4.777172588
HES1	1.553398126	6.118067642	2.561921701	0.012082556	0.0438489	-3.067307669
PAFAH1B1	1.5537001	4.110872305	2.618337467	0.010376592	0.041133873	-2.93698442
ETFB	1.569677469	8.962341048	1.973416948	0.051534701	0.088047275	-4.277196965
FXR1	1.570630941	5.840437228	2.678139276	0.008808904	0.039389826	-2.796208929
GTPBP6	1.572530644	8.43675561	3.788326739	0.000274496	0.012181799	0.270373437
CTSB	1.57373645	9.690221325	1.87976675	0.063395006	0.101248635	-4.443555505
MNAT1	1.575183031	4.670045349	3.082859406	0.002726019	0.028788954	-1.774637714
RARG	1.577436909	0.335722036	1.98552699	0.050148494	0.086916559	-4.255145298
WIPI1	1.577488171	6.53307033	3.021897583	0.003275758	0.029825904	-1.935985366
NME1	1.583454695	7.017049172	2.697158571	0.008357404	0.038684271	-2.750874196
PSMD1	1.592409673	6.991794486	2.952941189	0.00402042	0.032292944	-2.115358058
MBTPS1	1.592801126	5.384286585	2.750723755	0.007196642	0.038060621	-2.621747978
SDC1	1.592846646	8.857950136	2.464117289	0.015647114	0.0479969	-3.287458471
RFXANK	1.592973052	6.115350693	2.729739728	0.007632616	0.038187781	-2.672586519
OTUD4	1.597131182	3.071396617	1.915569847	0.058614363	0.095363445	-4.38083087
STMN1	1.600507316	7.166052641	4.832598321	5.55E-06	0.00122638	3.813787341
DFFA	1.602670404	4.800550882	2.044097388	0.043884337	0.079165911	-4.146761044
TFAM	1.606797042	4.126507941	3.758850386	0.000303932	0.013052198	0.178746473
ILF2	1.607857608	7.145551382	2.80120921	0.006239285	0.036399754	-2.49810568
AKAP17A	1.612430374	6.843668319	2.301567098	0.023682404	0.057004657	-3.636823299
CUL4A	1.61713657	5.903501417	2.532899828	0.013055201	0.045254127	-3.133402969
RAB14	1.628628551	4.886512343	3.068417703	0.002847902	0.029085678	-1.813093697
MAP2K2	1.630848161	5.203579544	2.504509304	0.014074244	0.046196988	-3.197433888
CIB1	1.632769605	7.671453489	2.150698823	0.034196582	0.068269745	-3.942194637
SSR2	1.637990209	9.306631596	3.680963096	0.000396863	0.014498194	-0.060899894
ZEB2	1.638131702	3.814537999	2.424331008	0.017348282	0.04928254	-3.374892044
TAF6	1.647891496	6.547483231	4.180516069	6.76E-05	0.006342835	1.536113757
FUT8	1.651045917	3.205422452	4.19535984	6.40E-05	0.006342835	1.585655342
ARL2BP	1.655032531	6.014917483	4.931560019	3.74E-06	0.00096284	4.176192652
HLA-DPB1	1.655746211	7.924495938	1.900565863	0.060579407	0.097659816	-4.407249172
SRF	1.658325182	0.838367047	3.002357012	0.003472643	0.030678318	-1.98715497
MTHFD1	1.659320366	7.220453176	1.726746965	0.087665127	0.127378088	-4.699291743
ALDH1A3	1.663922953	2.606820165	1.805447324	0.074368879	0.113041053	-4.570273389

PFKL	1.665987155	5.622699495	1.761770428	0.081524728	0.120609789	-4.642535477
KIAA0430	1.671824405	6.74231356	2.9620616	0.00391367	0.032013404	-2.091826145
NID1	1.674235369	-0.059058778	1.932751057	0.056430549	0.092909084	-4.350345085
PGK1	1.676243104	9.020440674	2.514365152	0.013712662	0.045787851	-3.175275793
EFNA1	1.676324794	8.447113504	2.223168386	0.028724214	0.062457996	-3.797807929
LMNB1	1.677479473	0.988726075	1.79760642	0.075613647	0.114382288	-4.583366776
GSN	1.681234841	7.591846387	2.005598439	0.047920959	0.084330231	-4.218325804
CDC40	1.683447608	2.056624493	3.882255044	0.000197761	0.010542713	0.565705979
ZBTB43	1.683976793	2.522800229	2.053759923	0.042918284	0.078496147	-4.128605918
NDRG1	1.684778314	10.5918563	1.839952743	0.069091683	0.10787954	-4.512027006
CTNNAL1	1.687347632	6.877498056	2.761297681	0.006985675	0.037723673	-2.596006948
GSTM3	1.688375269	9.724273167	2.547200102	0.012567466	0.044579376	-3.100915806
ELF3	1.6884935	4.256026841	1.946839249	0.054691631	0.091310218	-4.325161483
MANBA	1.691092093	3.972844433	1.763101616	0.081298523	0.120390342	-4.640357347
MX1	1.692730207	5.807689348	2.891898969	0.004806947	0.03424673	-2.271330749
S100A11	1.698172827	4.594440106	2.370266014	0.019923705	0.052653073	-3.491715241
HAX1	1.704379169	7.577252061	2.117329175	0.037007319	0.071371801	-4.007237291
CDC20	1.706438844	0.797785985	2.274603186	0.025320714	0.058513938	-3.692748674
GUK1	1.715133526	8.018482384	1.983873595	0.05033586	0.086948872	-4.258163281
GPX1	1.716795296	9.886986012	3.680091071	0.000398042	0.014498194	-0.06356253
LGALS3BP	1.717215391	4.387676346	2.165848096	0.032983017	0.067449397	-3.912365312
MPP1	1.719689128	7.631039525	2.873600574	0.005068952	0.034909058	-2.317566166
PYCR1	1.725743604	3.919704364	3.002917773	0.003466843	0.030678318	-1.985690273
NINJ1	1.731585492	7.386405422	2.699797387	0.008296452	0.038651569	-2.744562923
IDH3B	1.735984699	7.116313334	2.082338308	0.040167317	0.075089084	-4.074456262
EIF3B	1.736574957	7.298889026	2.144253286	0.03472457	0.068531403	-3.954829145
FSTL3	1.743319501	2.467900298	2.419225678	0.017578111	0.049428371	-3.386021829
IER2	1.746782017	8.576602434	2.361814443	0.020355611	0.053429158	-3.509769024
ERP29	1.74699159	7.129429297	1.759578381	0.081898347	0.121046697	-4.64611884
RANBP2	1.747661177	5.937816031	2.596982295	0.010994918	0.042361104	-2.986600699
ZYX	1.751564015	2.83063044	2.125814533	0.036274256	0.070363863	-3.990784653
CCT7	1.752108488	7.315415662	1.937967681	0.055781276	0.092311	-4.341039531
TRIM2	1.752417533	7.593020224	2.940932765	0.004165072	0.032357797	-2.146251423
SKP1	1.755766476	8.985529654	4.256665268	5.10E-05	0.005636822	1.791471345
NDUFA7	1.756681348	7.827087591	3.094331722	0.00263267	0.028662726	-1.743986106
PLEC	1.758461744	4.939928891	3.160584499	0.002149207	0.026401425	-1.565206738
VASP	1.762193038	4.525686577	2.476564632	0.015146419	0.047788498	-3.259851092
RARRES3	1.770495153	7.143877224	3.046812706	0.003039714	0.029718692	-1.870354643
EXT2	1.772871723	5.629107683	2.737538022	0.007467859	0.038060621	-2.653731446
EBP	1.77467521	7.743014767	2.08758961	0.039678715	0.074427931	-4.064432707

NDUFV2	1.775239339	8.119494193	2.442545662	0.016549987	0.048430961	-3.335017263
SRSF4	1.780515731	5.866105734	1.992562621	0.049357831	0.086148498	-4.242277372
MFAP2	1.781122537	0.326735743	1.942281675	0.055249137	0.091844264	-4.333326771
ERCC1	1.787317445	4.950703855	2.036828799	0.044623295	0.080117113	-4.160366997
XBP1	1.788351417	7.664758683	3.93366661	0.00016493	0.009807018	0.729486423
HEBP2	1.794906271	7.562218373	2.348337028	0.021061547	0.05417829	-3.538441731
ABCF1	1.797795545	5.874158763	1.735392764	0.086115109	0.125479697	-4.685379698
SFRP1	1.798828945	8.413671015	3.079180591	0.002756599	0.028788954	-1.784447502
ACOT8	1.799188428	4.2188768	1.750605986	0.083442377	0.122625395	-4.660742854
PDCD2	1.801378965	5.199911189	2.466357694	0.015555915	0.047973225	-3.282498291
ZNHIT1	1.803883019	8.315892328	2.883159002	0.004930496	0.034751544	-2.293444493
UQCRC2	1.804536962	7.984091602	2.531180321	0.01311498	0.04535964	-3.137298742
ITPR2	1.805252909	1.72331734	3.611176138	0.000502525	0.014940456	-0.272537207
PGRMC1	1.807721891	4.076527903	2.440384316	0.016642966	0.048430961	-3.339762358
PLOD3	1.813044623	6.558675958	3.159130221	0.002158866	0.026401425	-1.569163164
PFN1	1.816069918	4.801572698	2.857195399	0.005314943	0.035007172	-2.358812689
ITGB1BP1	1.819673159	6.14879169	2.00535798	0.047947136	0.084330231	-4.218768901
EIF2B1	1.826322662	5.774171299	2.327804349	0.022178745	0.055150654	-3.581847145
PTPN13	1.828466167	5.50897148	2.526718868	0.013271232	0.045695601	-3.14739615
PPP1R12A	1.829309085	1.68016175	2.115398686	0.037175877	0.07157398	-4.010972136
CHMP2A	1.829592077	7.912720473	2.216470614	0.029195662	0.062792188	-3.81133141
E2F6	1.851650012	4.571333225	2.381819394	0.019346452	0.052213278	-3.466944211
AMOTL2	1.856858093	7.806444942	3.9760602	0.000141846	0.009224523	0.86565309
BCAT2	1.858963061	4.814783839	2.348606777	0.021047208	0.05417829	-3.537869262
KIAA0355	1.86127472	6.12568469	2.182882972	0.031663338	0.065706739	-3.878599387
FRY	1.865039634	4.766377247	2.12447092	0.036389487	0.070498931	-3.993393787
ARHGDIB	1.866458387	6.277335583	1.8280477	0.070875942	0.109483141	-4.532238338
PHB2	1.867070143	8.71363242	2.218074263	0.029082171	0.06278676	-3.808096788
ACTN2	1.869683758	0.059853914	2.375848015	0.019642924	0.052448982	-3.479760311
RPL14	1.875343425	9.662245976	3.350377261	0.001183304	0.021180266	-1.036721415
CELF1	1.875784431	2.327882976	3.629454385	0.000472527	0.01485372	-0.217391265
TK2	1.879828786	4.577286397	1.923777565	0.057562376	0.094370556	-4.366298484
NID2	1.884323944	3.704147207	2.517079658	0.01361455	0.045756728	-3.169159827
RPS6	1.885310844	11.66356843	3.400339927	0.001007476	0.019697314	-0.893662781
GPKOW	1.888610776	5.648950306	2.342027781	0.021399412	0.054413637	-3.551814923
LARS2	1.891855514	5.267553753	2.444239372	0.01647745	0.048430961	-3.331296283
PREP	1.894719796	4.346078615	2.723159271	0.007774213	0.038233371	-2.688461972
GNS	1.895716121	1.159160931	1.998493337	0.048699624	0.085265706	-4.231398102
MTX2	1.896341633	6.890331176	2.524911687	0.013334999	0.04571155	-3.151481904
TCF4	1.898823546	5.319227891	1.910647757	0.059252983	0.096022129	-4.389518466

NCAPD2	1.901197544	2.954195271	2.552580897	0.012388234	0.044540024	-3.088651164
INTS3	1.905432636	3.598102037	1.746092168	0.084228164	0.123428191	-4.668073581
ACTN4	1.906544349	5.403386189	2.142696667	0.034853137	0.068531403	-3.957875321
IDH3G	1.907512942	5.71667038	1.774486755	0.079384966	0.118808478	-4.621666197
ABLIM1	1.920830183	8.82802121	3.088442848	0.002680209	0.028775027	-1.759731271
GOLGA3	1.923792902	6.129106536	2.771937484	0.006779109	0.037608532	-2.57002237
BMS1	1.927303421	3.967101709	2.094289286	0.039062803	0.073918107	-4.051611466
VAMP8	1.929402327	9.221236983	3.15435077	0.002190897	0.026423162	-1.582155745
PSME3	1.930072183	3.271233683	2.040010493	0.044298526	0.079727033	-4.154416644
RPL10A	1.930197168	11.08284242	3.144705627	0.002256889	0.026634733	-1.608328053
ARHGEF9	1.932616203	1.864782618	1.675599416	0.097309069	0.137891678	-4.780266183
PES1	1.933251093	3.613447977	1.84498982	0.068348131	0.107438167	-4.503439025
BCLAF1	1.943965463	4.796887745	2.668637293	0.009042674	0.039389826	-2.818756628
POLR2K	1.946241947	6.934710954	3.3424017	0.001213909	0.021180266	-1.059408228
FIBP	1.947032327	6.339134983	2.144815971	0.034678197	0.068531403	-3.953727527
SARS	1.94834468	6.340083477	2.281967405	0.02486365	0.057844288	-3.677532511
PAPPA	1.950211246	1.592985232	3.531328889	0.000656032	0.01635847	-0.5110362
PRPSAP1	1.953952611	6.271685363	2.246719863	0.027118977	0.061027567	-3.749967062
PPP1R12B	1.961419024	-0.664955505	1.869083518	0.064883442	0.103093321	-4.46206094
ARIH1	1.96599257	2.723988261	2.027716557	0.045564743	0.081249242	-4.177361946
MYL6B	1.968734962	6.98601388	5.253062881	1.00E-06	0.000388159	5.380412329
AURKA	1.968832514	1.016557681	2.781859717	0.006591519	0.037520949	-2.545715211
PLIN3	1.971896738	3.948658149	2.287280727	0.024538404	0.05779261	-3.66652696
LYN	1.973474766	3.588466975	2.176062844	0.032186059	0.066169743	-3.892146405
DBN1	1.975970455	6.2960142	3.337873647	0.001231614	0.021180266	-1.072270034
NFKBIE	1.979039592	4.699713321	3.204470485	0.001875992	0.025219854	-1.445139937
PHKB	1.983809244	6.739775308	3.067055925	0.002859653	0.029085678	-1.816712454
HMGCR	1.984543626	6.058977037	2.230123587	0.028241714	0.061992799	-3.783726244
BIRC5	1.989528558	1.380150628	4.260869791	5.03E-05	0.005636822	1.805657518
FOXO1	1.999507685	7.196674783	2.327625816	0.022188685	0.055150654	-3.582223089
MED1	2.003130064	2.05193706	2.050761431	0.043216096	0.078510088	-4.134248141
IRF2	2.003536975	2.533049416	6.135673554	2.27E-08	1.75E-05	8.864859241
RPL24	2.007428227	10.61957779	3.009264452	0.003401827	0.030678318	-1.969097492
BASP1	2.010772665	5.125039172	1.784742742	0.077693219	0.11650215	-4.604733164
LSM3	2.015904899	7.335876387	3.264478361	0.00155463	0.022969498	-1.278872968
FBXO7	2.017341702	7.289388377	2.392969872	0.018803471	0.051542848	-3.442937154
SLC20A1	2.02273425	5.949942436	2.701466391	0.008258109	0.038651569	-2.740568475
CNOT8	2.023132902	4.247018746	3.162806893	0.002134524	0.026401425	-1.559157849
TUBA1B	2.024694381	8.800980418	3.338304696	0.001229918	0.021180266	-1.071046225
KIF13B	2.03051009	7.560618968	3.277883518	0.00149026	0.022897772	-1.241403176

IK	2.034557107	6.927087922	2.328401052	0.022145553	0.055150654	-3.580590463
SIGMAR1	2.037658297	4.775641321	1.993803865	0.04921945	0.08607836	-4.240002877
CFB	2.037675306	5.024437405	2.377573682	0.019556835	0.052347533	-3.476059482
NRIP1	2.038212491	6.427369552	2.459652705	0.015830278	0.048274451	-3.29733126
G6PD	2.039579655	0.569965472	1.681116829	0.096228917	0.137115121	-4.771640794
BUB3	2.042674351	4.55385189	2.845154257	0.005502421	0.035007172	-2.388963149
SON	2.042816235	7.153994019	2.272792642	0.025434213	0.058688497	-3.696483007
RPS5	2.046678821	8.491550992	1.828348086	0.070830454	0.109483141	-4.531729861
QPRT	2.05177411	9.598730743	2.947597004	0.004084213	0.032292944	-2.129119375
ID2	2.05791006	6.914483841	1.816487586	0.072645108	0.111200641	-4.551747999
IDH3A	2.061292578	4.394348997	2.063939293	0.041920387	0.077153474	-4.109395874
GSS	2.061884175	7.649452225	1.805031034	0.074434535	0.113041053	-4.570969872
PSME1	2.062357574	9.24178038	2.641280372	0.009747502	0.040076729	-2.88329454
IGF2R	2.068246259	4.382160752	1.702314143	0.092169499	0.1325526	-4.738256378
ECD	2.068311604	5.23866828	3.421955586	0.000939297	0.019623685	-0.831272342
CD63	2.069093566	11.02765534	3.620348186	0.000487253	0.01485372	-0.244890318
OPTN	2.073810271	7.869837376	2.614716231	0.010479176	0.041434285	-2.94542229
SPTLC1	2.074035791	0.109048701	2.501112326	0.014200838	0.04622968	-3.205053649
COG2	2.074459052	5.472542912	2.267313544	0.025780415	0.059134305	-3.707767865
PPP2R5A	2.075633908	6.546697696	2.843514542	0.005528415	0.035028402	-2.393060792
IGBP1	2.076727024	6.387480192	2.691244214	0.008495498	0.039089405	-2.765000724
SF3A1	2.084494926	6.334904707	2.283155727	0.024790581	0.057844288	-3.675073089
NBAS	2.08560709	5.086664506	1.827123238	0.071016088	0.109483141	-4.533802736
KLHL20	2.090688653	3.562134192	1.806357001	0.074225573	0.113041053	-4.568750918
ICAM1	2.091131378	2.589781076	3.389098765	0.001044748	0.019697314	-0.925990214
PRCP	2.096129352	9.227797767	3.602372851	0.000517609	0.015098553	-0.29902429
PIGR	2.097060181	1.259155001	2.985339847	0.003652964	0.031374902	-2.031498314
VPS11	2.0989842	5.482142407	1.743433541	0.084693833	0.123993055	-4.672383116
VIM	2.099914118	8.048058532	2.374354229	0.019717717	0.052555179	-3.482961953
POLG	2.102482843	4.797338166	1.922025055	0.057785646	0.094492945	-4.369406219
MPZL2	2.102720336	3.489464462	2.00091002	0.04843357	0.085023509	-4.226956513
COL6A3	2.10541915	3.754738988	1.921048673	0.057910355	0.094492945	-4.371136515
CDK4	2.106193704	5.716885041	2.159392754	0.033495476	0.067691511	-3.92509899
FAM178A	2.1072447	1.073350252	2.65702799	0.009335927	0.03968189	-2.846212951
HSP90AB1	2.107884829	6.838118999	1.685846938	0.095310683	0.135954045	-4.76422508
DDX1	2.110638498	8.448827591	2.509324453	0.013896535	0.046169035	-3.186617752
LSM1	2.119236883	7.430925345	2.934187473	0.004248417	0.032458075	-2.163559671
AIMP1	2.121550157	6.700056865	2.196471282	0.030643907	0.06428152	-3.851495656
ATP6V0E1	2.122734254	8.001684193	1.678825795	0.096676246	0.137421437	-4.775225597
RPS24	2.122856644	9.982786426	2.743772185	0.007338488	0.038060621	-2.638625811

GAB2	2.123323474	3.847645531	2.872434884	0.00508608	0.034909058	-2.3205034
SLC31A2	2.123904726	6.00885614	2.771710169	0.006783463	0.037608532	-2.570578391
PSMA3	2.125130333	6.861724772	2.35568107	0.020674228	0.054003987	-3.522835396
RPL8	2.12718856	10.65083517	2.218836652	0.029028352	0.06278676	-3.80655829
COPS8	2.12722269	5.242867267	2.119892603	0.03678452	0.071086085	-4.002273186
SUPT7L	2.131991621	6.255968258	2.674446363	0.008899098	0.039389826	-2.804980053
TBCC	2.136033977	5.355179257	2.831084589	0.005729161	0.035220999	-2.424059744
PTPN1	2.141559412	4.574779979	2.332521391	0.02191755	0.055099562	-3.571905124
CHMP1A	2.143996848	5.636401542	2.26394375	0.025995391	0.059450998	-3.714696377
MARCKS	2.148833838	7.209563122	3.081144891	0.002740231	0.028788954	-1.779210737
MANF	2.148898148	8.270212327	4.460239652	2.37E-05	0.003333639	2.48847135
RRAGA	2.152348517	7.776351887	2.776173402	0.006698436	0.037520949	-2.559654209
CYB5B	2.156972361	3.46420192	2.965421577	0.003875007	0.032013404	-2.083142092
GPNMB	2.15797389	5.470424524	2.399613466	0.018486438	0.051106648	-3.42858689
ZNF207	2.158845043	4.741799116	2.345846429	0.021194352	0.054224389	-3.543724622
PSMD7	2.168388738	7.925361431	4.651615503	1.13E-05	0.002190325	3.161826102
FAU	2.178503786	10.6384785	2.82485909	0.005832196	0.035220999	-2.439543255
GLS	2.18957691	6.129188891	1.767227747	0.080600671	0.119815998	-4.633596332
RAB5B	2.19422517	-0.244873917	1.914372081	0.05876923	0.095363445	-4.38294684
PAFAH1B3	2.196823154	4.261110382	2.726168843	0.007709159	0.038233371	-2.681205319
PRSS23	2.201754988	5.774620585	2.373697372	0.019750685	0.052555179	-3.484369242
UBE2N	2.201885346	3.326678556	2.964849117	0.003881569	0.032013404	-2.084622211
ELF2	2.202252147	4.393358767	2.245011439	0.027232715	0.061194444	-3.753452505
TPST2	2.204838323	5.257785026	1.790165136	0.076810918	0.11562773	-4.595743993
CREBL2	2.209215667	3.536298495	2.670153106	0.009005008	0.039389826	-2.81516422
SS18	2.210948013	4.604100083	2.574808125	0.011672005	0.043273187	-3.037753008
IFNAR1	2.211591481	3.474482955	4.172141193	6.97E-05	0.006342835	1.508213031
UBE2L3	2.214177997	6.766543394	2.928949271	0.004314197	0.032458075	-2.176978415
CYBB	2.217959813	0.044096224	2.279526452	0.025014338	0.058066316	-3.6825809
UQCRC1	2.21892645	8.535047366	1.771973093	0.079804216	0.119054659	-4.625802528
SPEN	2.224485375	3.78637369	2.046814304	0.043610833	0.078790334	-4.141664015
DHFR	2.22659891	0.740681422	2.252947043	0.026707926	0.060454544	-3.737242761
CPD	2.229542883	2.095241883	3.550152846	0.000616284	0.016310363	-0.455164689
TRAPPC3	2.229562889	5.518934694	1.678650417	0.096710558	0.137421437	-4.775499823
KAT7	2.231557457	2.694338893	1.701790451	0.092268077	0.132571048	-4.739085869
PLK2	2.235363328	4.663149669	4.36873652	3.36E-05	0.00432343	2.17264965
BACH1	2.23995071	4.865886463	2.668548612	0.009044882	0.039389826	-2.818966745
SERPINA1	2.241386761	6.88621974	2.560413275	0.012131455	0.043923252	-3.070758905
SEMA3C	2.243460859	2.776672691	1.799388995	0.07532915	0.114063531	-4.580394723
HMGCL	2.247437272	7.37347448	1.668881903	0.098637447	0.139136399	-4.790731854

SLC25A12	2.257267504	5.593358146	2.337921601	0.021621871	0.054530852	-3.560501475
ANAPC5	2.25915893	8.399550887	2.442018694	0.016572614	0.048430961	-3.336174522
HDAC4	2.260249663	3.66445144	2.397075	0.018607008	0.051185827	-3.434074115
FARP1	2.268368678	6.32220941	1.823979915	0.071494338	0.110089886	-4.53911647
MSH6	2.269065415	6.110563079	3.575065714	0.000567174	0.015942746	-0.380883335
KRAS	2.26938331	2.660715241	2.34471666	0.021254838	0.054224389	-3.54611939
NFIL3	2.274210619	6.09873104	2.238185538	0.027691335	0.061598315	-3.767354868
RPS6KB1	2.274800819	3.637750434	2.668789898	0.009038876	0.039389826	-2.818395038
C11orf49	2.277787348	5.34976093	2.282221025	0.02484804	0.057844288	-3.677007698
PSMB1	2.280466649	8.342822849	2.315137492	0.02289381	0.055904864	-3.608457167
NAA10	2.280657416	4.89081844	1.846482822	0.068129029	0.107258125	-4.500889363
HAT1	2.281154355	6.509969169	2.619815432	0.010334985	0.041099266	-2.933537749
APPBP2	2.281719017	5.32163578	2.803962433	0.006190591	0.036399331	-2.491309083
UPP1	2.282134363	2.938210338	2.097126218	0.038804498	0.073532719	-4.046171234
NPEPPS	2.282331635	7.736725934	3.537120039	0.000643552	0.016310363	-0.493870888
LIMK2	2.286269046	5.902073775	2.444607048	0.016461741	0.048430961	-3.330488224
GALE	2.28696006	4.935221007	1.844284296	0.068451872	0.107438167	-4.504643216
GRSF1	2.289051962	6.521692726	2.909365208	0.004568536	0.033159424	-2.226973611
LRP10	2.298370855	7.555451874	3.185141705	0.001992065	0.025452329	-1.498181429
GNG11	2.304375988	7.574973699	2.847800204	0.005460711	0.035007172	-2.382346825
DHCR7	2.304534037	5.608326796	3.67228344	0.00040875	0.014498194	-0.08738198
ERBB3	2.307839559	5.150845172	1.897016713	0.061052267	0.098319589	-4.413470448
ZNF217	2.30810146	5.023671556	2.975626038	0.003759739	0.03193712	-2.056719122
NUBP1	2.308234557	4.812545291	1.755008481	0.082681796	0.121855154	-4.653575961
CCT4	2.308354073	9.832530197	2.921166975	0.004413662	0.032648426	-2.19687826
ATP5O	2.310431546	10.66593173	2.749414258	0.007223169	0.038060621	-2.624930056
TOMM40	2.312514396	5.019202913	1.880311183	0.063319927	0.101233307	-4.442609841
SRP14	2.316433135	11.24605355	3.122964912	0.002412488	0.027424312	-1.667089389
GPX4	2.317732447	8.657971073	1.989350233	0.04971751	0.086363225	-4.248157863
PTPRM	2.320262908	5.846077715	1.990463895	0.049592565	0.086243088	-4.246120209
RREB1	2.322216883	6.154617847	2.268036736	0.025734483	0.059116659	-3.706279748
CBX3	2.323269239	7.923253163	3.12784964	0.002376682	0.027217412	-1.653914944
PEX19	2.323372239	5.139954964	2.00981699	0.04746369	0.083957512	-4.210544353
RPL12	2.32477818	11.27544188	2.934653632	0.004242608	0.032458075	-2.162364562
TRAK2	2.326762276	4.632148237	2.450687158	0.0162039	0.048430961	-3.317110394
TERF2IP	2.330994457	7.12193719	2.295389592	0.024049257	0.057306381	-3.649687369
RHOG	2.34152706	4.101363844	2.466675854	0.015543002	0.047973225	-3.281793577
ERCC5	2.346711644	6.574865651	2.157209324	0.033670369	0.067849344	-3.929398275
MAPKAPK3	2.347973311	6.51829123	1.884385551	0.062760433	0.100651068	-4.435524791
USP8	2.349490181	4.235424753	3.149790604	0.00222187	0.026423162	-1.594537744

ARMC8	2.35071402	4.767042329	3.107675697	0.002527831	0.028176118	-1.708219828
GPRC5B	2.350941668	6.181653971	2.070502375	0.04128764	0.076627481	-4.096964778
SOD1	2.35245881	11.23128889	2.845539474	0.00549633	0.035007172	-2.388000212
CELF2	2.352745071	3.426491598	2.3291181	0.022105724	0.055150654	-3.579079956
C21orf33	2.353540912	9.354600867	2.023332042	0.046023769	0.081973211	-4.185514718
TMBIM6	2.356715804	8.032851088	2.669363344	0.009024615	0.039389826	-2.817036135
SAMM50	2.360450463	7.818984512	1.973875403	0.051481635	0.088047275	-4.276364386
HCCS	2.363946828	4.698207558	2.155139455	0.033836898	0.067849344	-3.933470353
PLEK	2.365015683	0.045299504	1.902722603	0.060293571	0.097300481	-4.403463424
MNT	2.372549779	5.967737283	2.192207139	0.030960685	0.06464583	-3.86001724
LEPROTL1	2.380444761	6.783265166	3.48152909	0.000773196	0.01832181	-0.65778121
PAPD7	2.381025751	4.463501867	1.771654454	0.079857491	0.119054659	-4.626326471
AP1S2	2.381149977	4.293295408	2.706901566	0.008134361	0.038495667	-2.727546015
RPL23A	2.382033974	11.17470106	3.168498107	0.002097348	0.026401425	-1.54365229
COX7C	2.38279646	10.91939225	2.666054952	0.009107172	0.039420389	-2.824872687
FNDC3A	2.39053516	7.270438306	2.670714366	0.008991098	0.039389826	-2.813833624
ATP6V1B2	2.391498893	6.995015827	1.843151539	0.068618711	0.107540831	-4.506575721
RUFY3	2.394856787	0.827691813	2.091682516	0.039301459	0.07409763	-4.056604473
USP9X	2.399098012	7.675972912	3.027973753	0.003216677	0.029825904	-1.920019628
VPS72	2.400041379	5.421042304	2.006175575	0.047858179	0.084330231	-4.217262109
PCGF2	2.400120244	2.097176546	1.90594336	0.059868836	0.096817176	-4.397802656
GNL2	2.401194301	4.702279841	2.399071733	0.01851211	0.051106648	-3.429758342
CUL5	2.401425688	5.448812219	2.22980613	0.028263581	0.061992799	-3.784369827
SKP2	2.404517881	-0.634112511	2.149829913	0.03426735	0.068269745	-3.943899853
CNN3	2.415705321	6.042342163	3.410572554	0.00097464	0.019697314	-0.864165121
IVNS1ABP	2.416275325	7.676961315	2.190306022	0.031102835	0.064804559	-3.86381172
MAP1A	2.422042018	0.955601347	1.990869518	0.049547124	0.086243088	-4.245377787
ODC1	2.422715967	8.606640193	2.541931971	0.012745203	0.044579376	-3.112902139
PJA2	2.423515801	5.8634801	2.091884263	0.039282944	0.07409763	-4.056218247
TRIOBP	2.424274331	5.491642897	1.967746222	0.052194945	0.088967348	-4.287480708
CPSF6	2.424996773	-0.231127113	2.251408307	0.026808984	0.060594576	-3.740389841
RBBP7	2.427062638	7.997830195	2.83616816	0.005646266	0.035220999	-2.41139543
MAP2K1	2.427672678	4.81178073	2.111792884	0.037492493	0.071883811	-4.017939936
TUBB2A	2.430270967	8.193671604	2.439856009	0.016665764	0.048430961	-3.340921666
MTRR	2.43173505	3.073682811	3.540106713	0.000637205	0.016310363	-0.485010061
SSR4	2.432309601	7.983926883	2.525459836	0.013315629	0.04571155	-3.15024289
KTN1	2.435584312	8.298897606	3.396601998	0.001019728	0.019697314	-0.90442137
UQCRQ	2.437986956	10.80148726	2.855765776	0.005336891	0.035007172	-2.36239789
SAFB	2.438119765	4.507763687	2.443366542	0.016514796	0.048430961	-3.333214118
PTDSS1	2.438688311	8.312102733	2.66145097	0.009223199	0.039567308	-2.835764415

PSMA2	2.438769833	7.60678214	2.350741994	0.020934009	0.05417829	-3.533335806
EIF4A3	2.439124935	7.570729271	3.334062367	0.001246704	0.021180266	-1.083085503
TP53BP2	2.439682249	4.011823565	2.066024857	0.041718419	0.07705696	-4.105449488
SLC16A1	2.448698066	3.85581063	2.874494274	0.005055857	0.034909058	-2.315313611
CLN5	2.451774322	6.114572903	3.276685668	0.001495909	0.022897772	-1.244756218
F2R	2.453447247	2.449104086	2.37051934	0.019910886	0.052653073	-3.491173229
GADD45A	2.455675989	7.388966637	1.663858491	0.099640411	0.14042304	-4.798532437
MEF2D	2.456928081	1.164868964	2.465955207	0.015572264	0.047973225	-3.283389671
CSNK2A2	2.457923816	3.872061763	1.854530784	0.066958065	0.105629763	-4.487112748
SSBP2	2.458508518	3.616170338	1.882148917	0.063067052	0.100933398	-4.439415892
COX6B1	2.459861975	8.951668328	2.852178358	0.005392333	0.035007172	-2.371387886
CSE1L	2.465667266	7.169780456	2.577108621	0.01160005	0.043213682	-3.032463545
PPIP5K2	2.465891395	5.325672648	2.636643653	0.009871782	0.040162565	-2.894177215
NDUFA5	2.466534503	8.652287429	2.422625485	0.017424761	0.04928254	-3.378612411
WDR77	2.468262568	4.512021175	1.870747224	0.064649737	0.102827669	-4.459185462
PSMD4	2.468683698	7.537020017	1.866959661	0.065182814	0.10346266	-4.465728298
SLC25A5	2.46957693	10.52306059	2.562453359	0.012065364	0.0438489	-3.066090835
ALG8	2.470314491	6.156747636	3.189753123	0.001963779	0.025452329	-1.48554977
NDUFAB1	2.47114066	8.018073853	2.007940074	0.047666675	0.084124064	-4.214008309
MRPL19	2.471996545	7.931648132	2.950295563	0.004051884	0.032292944	-2.1221731
PLXNA3	2.473010329	-0.775155384	2.052774104	0.043016001	0.078496147	-4.130461745
MTHFD2	2.473380051	3.899395613	2.068941108	0.041437411	0.076742448	-4.099925198
COIL	2.476090227	0.76473162	2.671328823	0.008975891	0.039389826	-2.812376641
AHR	2.476701346	4.863430727	2.447648706	0.016332298	0.048430961	-3.323799389
NUDT21	2.477907795	5.838411749	2.917109025	0.00446636	0.032725083	-2.207237554
MORC2	2.481671138	6.898207167	2.611319144	0.010576243	0.041711406	-2.953328839
TRAK1	2.48331267	5.244567177	1.956661716	0.053506302	0.089940802	-4.30750419
M6PR	2.485518983	3.911358578	3.028107372	0.003215389	0.029825904	-1.919668241
EIF4G3	2.486040596	4.236134156	2.742734386	0.007359881	0.038060621	-2.641142435
ENC1	2.48802536	2.280054356	2.111449218	0.037522791	0.071883811	-4.018603473
DNM1L	2.488653495	3.760173734	2.458797432	0.015865584	0.048283845	-3.299220802
ZBTB40	2.491865074	-1.042023731	1.937586926	0.055828451	0.092311	-4.341719509
RBM15B	2.493602562	4.928795977	2.808058932	0.006118782	0.036243819	-2.481186224
PSMB3	2.49927432	6.574359971	2.13155544	0.035785456	0.069677979	-3.979619805
TOPORS	2.499753946	4.150689129	2.703800261	0.008204764	0.038651569	-2.734979314
NACA	2.500528655	10.10364473	2.40745602	0.018118319	0.050531558	-3.411602173
CHTOP	2.503648294	5.247653586	1.883082326	0.062938939	0.100832745	-4.437792546
RPS6KB2	2.504522846	1.922912624	1.738069735	0.085639771	0.125140914	-4.681059032
AZIN1	2.50852322	4.908180545	2.99341425	0.003566351	0.03094564	-2.010483423
ZBED1	2.511098792	4.960977661	1.694543267	0.093641128	0.134169772	-4.750540412

RPL29	2.511934307	7.464641625	1.958741526	0.053258139	0.089940802	-4.303755016
WBP4	2.512924152	4.656591367	3.703338821	0.000367714	0.014498194	0.007577575
ZBTB5	2.514148319	4.93775572	2.23925555	0.027618998	0.061598315	-3.765178052
PNPLA6	2.521674539	3.174584762	2.008912263	0.047561443	0.084034275	-4.212214446
SEC24D	2.524401322	2.57959566	2.665208842	0.009128395	0.039420389	-2.826875539
PLK1	2.525008956	-1.189990303	2.064347147	0.041880824	0.077153474	-4.108624401
TMX2	2.527877961	6.492107543	2.299652502	0.023795571	0.057004657	-3.640813527
PPP1R7	2.531345402	6.751597303	2.483164572	0.014886799	0.047284514	-3.24516405
DPF2	2.533417046	5.927078418	3.345558145	0.001201709	0.021180266	-1.050434538
EAPP	2.533607053	7.332715009	3.45005066	0.000857162	0.019205401	-0.749733948
STX7	2.535056438	4.438393971	2.841428644	0.005561646	0.03509512	-2.398270629
FZD7	2.536222065	4.209833993	2.046830366	0.043609221	0.078790334	-4.141633865
RPS17	2.537078097	11.29082539	2.950726809	0.00404674	0.032292944	-2.121062564
HSPG2	2.537406297	1.244123304	1.77350306	0.079548818	0.118938561	-4.623285555
MDH1	2.539436779	10.61458318	2.763473636	0.006942969	0.037723673	-2.590699604
R3HDM2	2.540508563	5.904819796	2.684145252	0.008663989	0.039313143	-2.781922193
BUB1B	2.542027763	-0.076938305	2.1986237	0.030485084	0.064038281	-3.847188591
PSMD6	2.545258203	6.836129604	2.385986936	0.019141901	0.052009453	-3.457982933
MTR	2.552778209	7.357859281	2.873722564	0.005067163	0.034909058	-2.317258726
RAD23B	2.558037323	7.351823254	4.028351004	0.000117616	0.008474605	1.034978177
ECHS1	2.563297885	10.91067801	1.977471295	0.051067022	0.087819372	-4.26982794
CDC42BPA	2.564080845	5.411595205	2.06830566	0.041498503	0.076742448	-4.101129536
ITGA6	2.564116655	7.28704428	2.68228445	0.008708654	0.039367191	-2.786351468
PARP4	2.564245914	5.622196364	2.492965326	0.014508622	0.046720644	-3.223291821
COL15A1	2.566582206	1.343668642	2.225634242	0.028552332	0.062171697	-3.792819969
FAM189B	2.567017076	4.830466811	2.09950574	0.03858898	0.073471137	-4.041603032
BRD8	2.573422175	6.789986682	2.053242928	0.042969506	0.078496147	-4.129579273
PEX3	2.57637228	4.860252447	2.794978431	0.006350782	0.036740226	-2.513466486
GLUD1	2.577334486	7.491227098	2.735589214	0.007508725	0.038060621	-2.658447586
KIAA0196	2.577725521	5.809730549	2.546582023	0.012588203	0.044579376	-3.102323196
PSMC2	2.581464767	6.974495931	2.299263657	0.023818613	0.057004657	-3.641623563
KIF3C	2.583582953	-1.04076018	2.036109543	0.044696995	0.080117113	-4.16171097
SLC25A3	2.585467012	10.75272212	2.46232389	0.015720461	0.0481264	-3.291426179
LAMC2	2.586613491	3.65372446	3.8904544	0.000192136	0.010542713	0.59172636
PLCB4	2.590771527	2.296884534	2.492248601	0.014535983	0.046720644	-3.22489385
MGST2	2.592345866	7.960489379	2.507433709	0.013966074	0.046169035	-3.190867013
TDG	2.593471436	6.571558774	2.02004011	0.046371008	0.082496638	-4.191625356
NDST1	2.595927062	0.002182846	2.543880978	0.012679185	0.044579376	-3.108470136
POLR2B	2.598547138	5.359438207	2.159530255	0.033484489	0.067691511	-3.924828113
MMD	2.598762101	4.673267637	2.030378549	0.045287971	0.080942431	-4.172404299

TTC1	2.60150205	4.919380678	1.940863674	0.055423573	0.091846647	-4.335863671
FURIN	2.611874307	2.250708238	1.691453612	0.094231539	0.134890703	-4.755409876
GUSB	2.617009671	8.481724668	2.656753853	0.009342955	0.03968189	-2.846860068
CDYL	2.619727657	1.922948768	3.24650271	0.001645039	0.023120273	-1.328931022
MLEC	2.628781225	7.867957925	2.736016995	0.007499737	0.038060621	-2.657412592
PLA2G2A	2.632682011	-0.925405278	2.06204357	0.0421047	0.077400554	-4.112979908
PTTG1IP	2.632728905	8.869968517	2.310410551	0.023165827	0.056311899	-3.618354558
DAD1	2.633470513	9.02525663	3.006067525	0.003434434	0.030678318	-1.977459082
BLZF1	2.637966093	4.409156911	3.304314052	0.001370592	0.021766834	-1.167177495
KRR1	2.638613959	5.961546318	2.11013567	0.03763879	0.072016794	-4.021138722
RAD21	2.640270338	7.625765747	2.734363049	0.007534542	0.038066675	-2.661413479
SEC63	2.653664866	5.937667554	3.307864185	0.001355223	0.021766834	-1.157172522
HADH	2.655390789	9.619349036	2.143420523	0.034793299	0.068531403	-3.956459039
ERH	2.659625319	8.080121725	2.459628598	0.015831272	0.048274451	-3.297384528
ADRBK2	2.663242793	-0.481946153	2.177584761	0.032068766	0.066169743	-3.889126662
PRDX4	2.664008048	7.920649433	2.672873516	0.008937767	0.039389826	-2.808712655
PMPCB	2.665712833	8.445011376	2.423407473	0.017389658	0.04928254	-3.376906893
RPL32	2.666608607	11.49631457	2.736313864	0.007493506	0.038060621	-2.656694253
GCLC	2.667165046	5.915513568	2.164068488	0.033123606	0.067469163	-3.91587913
NDUFA1	2.671650469	9.127287128	2.43667126	0.016803787	0.04874044	-3.347905609
RPLP0	2.68376754	10.67468438	1.908584014	0.059522486	0.096357868	-4.393154914
LEPROT	2.685070918	8.630316151	2.969029686	0.003833882	0.032013404	-2.073807831
COX4I1	2.700037026	9.777836762	1.705962473	0.091485132	0.132059771	-4.732471067
RNF114	2.704497593	5.970899442	2.728412289	0.007660989	0.038206093	-2.675791572
ADAM12	2.705537341	0.083330294	2.706996613	0.008132212	0.038495667	-2.72731809
RNF44	2.712772507	4.427156376	2.708068919	0.008108004	0.038495667	-2.724746212
NOTCH2	2.717406631	6.929531931	2.510133299	0.013866882	0.046169035	-3.184799107
C1orf123	2.717711338	5.977992546	2.341157556	0.021446388	0.05444354	-3.553656986
SPARCL1	2.718652909	7.175881622	2.769870814	0.00681879	0.037649464	-2.575076125
FSCN1	2.719101254	0.11236837	1.791500097	0.076594979	0.115415046	-4.593527034
ARG2	2.719207544	6.592942241	2.244087792	0.027294381	0.061243995	-3.755335903
RFC4	2.721259233	4.68776904	3.044988117	0.00305645	0.029718692	-1.875175606
ASF1A	2.721667782	3.862871985	2.747624747	0.007259565	0.038060621	-2.629276522
NPC1	2.724945832	3.350356773	1.829146683	0.070709639	0.109483141	-4.530377661
TBCA	2.725186969	10.15314919	2.760749006	0.006996482	0.037723673	-2.597344665
ACSL4	2.726752953	4.578033432	2.641409845	0.009744052	0.040076729	-2.882990425
IKBKAP	2.727296467	5.618566912	2.178543533	0.031995065	0.066169743	-3.887223326
NDUFAF1	2.730291779	7.388942117	2.827034938	0.005795993	0.035220999	-2.434134883
KIAA0020	2.731632607	6.462120149	3.338540835	0.00122899	0.021180266	-1.070375744
NUPL2	2.732309263	2.455357044	2.499437715	0.01426362	0.04622968	-3.208806686

ZNF185	2.734256562	3.352935057	1.871610794	0.064528709	0.102740869	-4.457691976
HNRNPU	2.73557904	6.3980563	3.325803154	0.001280002	0.021228506	-1.106490571
RPS18	2.736683316	11.70799454	2.57577568	0.011641693	0.04326456	-3.035528829
NT5E	2.742148951	1.906658875	2.103409858	0.038237616	0.072981919	-4.034097801
EIF2AK2	2.745333925	5.086482595	2.856556324	0.005324744	0.035007172	-2.360415541
MYBL2	2.745731511	1.174938059	2.543925339	0.012677686	0.044579376	-3.108369225
CTSH	2.75144612	10.88825579	2.406339879	0.018170308	0.050531558	-3.414022363
GSTA4	2.752613044	5.955162327	1.822740033	0.071683719	0.110271672	-4.541210141
PDE6D	2.757815764	3.682130464	2.029602128	0.045368547	0.080992811	-4.173850902
RELA	2.771576741	4.807194631	1.763364204	0.081253964	0.120390342	-4.639927513
IL13RA1	2.775610056	5.168317999	3.323183486	0.001290737	0.021228506	-1.11390491
RPS21	2.77574165	11.29078565	2.803167993	0.006204605	0.036399331	-2.493270803
SSFA2	2.775828055	6.742414759	2.867695012	0.005156273	0.03496315	-2.332436543
TACSTD2	2.77609487	8.271113151	1.956822604	0.05348707	0.089940802	-4.307214294
SNRPD3	2.776437063	8.439773302	2.982990711	0.003678526	0.031419893	-2.037603681
CSTB	2.777451564	7.304766063	2.925688553	0.004355618	0.032458075	-2.185321547
COX11	2.778494406	7.06694043	2.927599796	0.004331295	0.032458075	-2.18043221
UBAP2L	2.778997944	3.900600228	1.715056912	0.089797232	0.129865782	-4.717999293
ICMT	2.783217865	4.655927408	1.850674324	0.067517054	0.106403023	-4.493721189
SGK1	2.784387631	8.644581654	1.957241687	0.053437002	0.089940802	-4.306459071
RNF14	2.794631598	4.66834674	2.229717645	0.028269679	0.061992799	-3.7845492
IGF1R	2.797392809	1.338099003	2.139821509	0.035091694	0.068822491	-3.963496549
GCA	2.798470647	6.501452711	2.899382801	0.004703426	0.033820916	-2.252351649
ANXA2	2.79890927	7.837625217	2.114097274	0.037289883	0.071704178	-4.0134882
NUCB2	2.800680871	5.995357876	3.402328612	0.001001014	0.019697314	-0.887935249
CSF3R	2.807631973	-0.273584556	2.370913801	0.019890939	0.052653073	-3.490329144
ARHGAP32	2.80842853	3.330362588	1.947101342	0.054659716	0.091310218	-4.324691388
TRIP12	2.810974835	6.870977519	3.193657236	0.001940125	0.025452329	-1.474844362
TCEB2	2.815288477	6.036643139	1.718176034	0.089224236	0.12915793	-4.713019368
CRCP	2.816961465	3.675217504	2.193193397	0.030887164	0.064616448	-3.858047588
CNOT4	2.817529419	5.5800642	2.623313931	0.010237099	0.041001436	-2.925372527
LBR	2.81789485	8.012952198	2.706548142	0.008142356	0.038495667	-2.728393475
CTNNB1	2.821853478	3.082149004	1.81405641	0.073021817	0.111553092	-4.555836451
ATP6V1C1	2.822007613	6.567859534	3.021496651	0.003279692	0.029825904	-1.937037948
WIPF1	2.823322022	0.868569967	1.755717926	0.082559768	0.121791413	-4.652419477
HDAC1	2.826279159	7.389424549	2.31612131	0.022837551	0.055904864	-3.606394983
MFAP1	2.830645316	6.535896854	2.946683074	0.004095216	0.032292944	-2.131470723
HLA-DMB	2.831735447	5.036268436	1.963953308	0.052640558	0.089529486	-4.294344002
SATB1	2.839934446	5.940239686	2.913088471	0.004519144	0.032955643	-2.217489777
ETHE1	2.84378979	5.715122619	2.712327253	0.008012515	0.038470024	-2.714524344

TGOLN2	2.845356599	5.30363663	2.2408119	0.027514079	0.061567943	-3.762010188
CALU	2.846449619	3.808152578	2.379424331	0.019464883	0.052327856	-3.472088006
ARHGEF11	2.849073327	-0.553450493	2.393865344	0.018760459	0.051516287	-3.441004945
TMEM59	2.852213872	9.904207505	3.097431549	0.002607961	0.028595092	-1.735688461
TBP	2.860429608	4.112897926	2.162391131	0.033256594	0.067650914	-3.919188685
H2AFZ	2.860874775	7.03870917	2.640327908	0.009772915	0.040076729	-2.885531357
FAIM2	2.866247675	-1.178441281	2.191883573	0.030984838	0.06464583	-3.86066326
RPP30	2.868202539	3.739168175	2.234068993	0.02797118	0.061864727	-3.775720883
NSMAF	2.870951666	5.23408428	1.98185181	0.050565784	0.087248552	-4.261850583
RNPS1	2.874240796	6.11446886	1.748700642	0.083773329	0.12299484	-4.663839401
TAF10	2.878433286	6.497068137	2.100844468	0.038468184	0.073331457	-4.039030893
TRIP4	2.879560697	6.482192962	2.328700688	0.022128902	0.055150654	-3.579959311
FAM20B	2.881414504	1.439985859	2.052526086	0.043040616	0.078496147	-4.130928517
TPMT	2.890015498	5.804509534	2.603173426	0.010812316	0.042118096	-2.972251939
RPS15	2.891821352	10.88246637	2.546781399	0.01258151	0.044579376	-3.101869241
YME1L1	2.897201813	6.628800998	2.827526978	0.005787835	0.035220999	-2.432911371
LAMC1	2.899674479	5.600539401	3.257788609	0.001587719	0.022969498	-1.297527467
MYO6	2.902036296	7.284250288	2.650214121	0.009512038	0.039795714	-2.862280772
RPL37A	2.904448311	11.15932704	1.750828409	0.083403814	0.122625395	-4.660381169
CLTC	2.905116482	9.622368169	2.441188222	0.016608329	0.048430961	-3.337997861
GABARAP	2.908794651	8.195547751	2.164638965	0.033078482	0.067466138	-3.91475301
SLC9A6	2.913335784	5.216884037	3.052744074	0.002985895	0.029718692	-1.854666675
TMED10	2.914768624	7.534049413	2.894787031	0.004766752	0.034117585	-2.264011349
TOMM70A	2.914943329	4.059088794	2.651018577	0.00949109	0.039795714	-2.860385597
KHDRBS1	2.916367692	5.32669633	1.975432324	0.051301769	0.08802723	-4.273535619
RPL6	2.935979297	10.46774909	2.519140866	0.013540473	0.045756728	-3.164511991
AMD1	2.936263969	6.580068325	2.527274251	0.01325169	0.045695601	-3.146140011
PALLD	2.938644467	7.969324585	2.210869997	0.029595066	0.063459047	-3.822611724
TERF1	2.93866632	5.652375577	2.452578144	0.016124447	0.048430961	-3.312943852
NUP88	2.942004148	5.455782489	2.185588373	0.031458045	0.065456444	-3.873215076
TUBG1	2.944536951	4.459190212	1.83330328	0.070083584	0.108893689	-4.523330822
BNIP3	2.948770092	9.360864945	2.522114571	0.01343424	0.045756728	-3.157800788
EIF3M	2.949528113	8.623059358	2.603598894	0.010799869	0.042118096	-2.971264791
RCN1	2.950002996	6.110643948	3.259903399	0.001577188	0.022969498	-1.291633545
GOT2	2.951142372	7.493881307	1.973727244	0.051498779	0.088047275	-4.27663347
SDC4	2.952411345	7.970776819	2.209184583	0.029716192	0.063630517	-3.826001372
SLC34A2	2.957508516	0.317227997	2.674438217	0.008899298	0.039389826	-2.80499939
APOD	2.965281792	-0.349386798	1.999686094	0.048568157	0.08513194	-4.229206561
PPP1R11	2.970860575	5.018791311	1.676234368	0.097184264	0.137841167	-4.779274911
RAB11FIP2	2.972535223	4.213968316	2.117129776	0.037024699	0.071371801	-4.007623203

TPD52L2	2.974577161	3.537368401	1.704481059	0.091762519	0.132213284	-4.734821603
FOXJ2	2.975889232	3.713936335	1.962737223	0.052784113	0.089674988	-4.296541952
EIF2B2	2.977465119	6.314638505	2.284977917	0.024678904	0.057808462	-3.671299578
GALNT1	2.980122124	4.355033767	2.70213926	0.008242697	0.038651569	-2.738957503
RAE1	2.981279327	4.614000669	1.929405502	0.056850303	0.093302089	-4.356300887
NUP133	2.981434142	6.103328853	2.600728864	0.010884086	0.042172424	-2.977921012
HMGN4	2.982993201	6.476153	3.022720402	0.003267699	0.029825904	-1.933824838
UBE4A	2.983281961	7.562640976	2.270577908	0.025573658	0.058834635	-3.701047421
BAZ2B	2.983893868	4.509000162	2.247651969	0.027057098	0.060977076	-3.748064434
NDUFS2	2.983931703	7.537307895	1.816008737	0.072719177	0.111200641	-4.55255367
USPL1	2.984500981	2.958993846	1.960406572	0.05306017	0.08984778	-4.300750893
DPYSL2	2.984685265	8.013257642	3.503032603	0.000720367	0.017401367	-0.594607388
CLIP1	2.986731522	6.599434658	2.845963241	0.005489638	0.035007172	-2.386940782
COG5	2.988717907	3.870285006	2.494294089	0.014458018	0.046664084	-3.220320709
DUSP14	2.989792336	4.012445248	2.317772156	0.022743424	0.05590037	-3.602932905
AGAP1	2.995957046	3.765686931	2.129713523	0.035941657	0.069806284	-3.983204892
CCNH	2.996623722	6.018396976	2.540914712	0.012779783	0.044599423	-3.115214208
PWP1	2.997599438	4.770935248	2.825750285	0.005817343	0.035220999	-2.437328484
PSMD12	2.998842559	5.162597751	2.295263134	0.024056818	0.057306381	-3.649950387
BMP1	2.999893723	0.963555277	1.975453054	0.051299378	0.08802723	-4.273497941
